# Mitonuclear Interactions and the Origin of Macaque Societies

**DOI:** 10.1093/gbe/evad010

**Published:** 2023-02-09

**Authors:** Jianlong Zhu, Ben J Evans

**Affiliations:** Biology Department, McMaster University, Hamilton, Ontario, Canada; Biology Department, McMaster University, Hamilton, Ontario, Canada

**Keywords:** *Macaca*, female philopatry, dispersal, natural selection, behavior

## Abstract

In most eukaryotes, aerobic respiration requires interactions between autosomally encoded genes (*N*_interact_ genes) and mitochondrial DNA, RNA, and protein. In species where females are philopatric, contrasting distributions of genetic variation in mitochondrial and nuclear genomes create variation in mitonuclear interactions that may be subject to natural selection. To test this expectation, we turned to a group with extreme female philopatry: the macaque monkeys. We examined four genomic data sets from (1) wild caught and (2) captive populations of rhesus macaque, which is the most widely distributed nonhuman primate, and (3) the stump-tailed macaque and (4) a subspecies of longtail macaque, both of whose mitochondrial DNA is introgressed from a highly diverged ancestor. We identified atypically long runs of homozygosity, low polymorphism, high differentiation, and/or rapid protein evolution associated with *N*_interact_ genes compared with non-*N*_interact_ genes. These metrics suggest a subset of *N*_interact_ genes were independently subject to atypically pervasive natural selection in multiple species. These findings suggest that natural selection on mitonuclear interactions could have influenced several aspects of macaque societies including species diversity, ecological breadth, female-biased adult sex ratio and demography, sexual dimorphism, and mitonuclear phylogenomics.

**Significance**

Endosymbiosis between the mitochondria and eukaryotic cell may have contributed to a myriad of important biological innovations (the nucleus, sex, the sequestered germline, speciation, and more), yet it remains unclear whether ongoing natural selection on mitonuclear interactions is atypically strong, persistent, or prevalent. To address this question, we searched for and found signatures of natural selection on mitonuclear interactions in several species of macaque monkey that share an extreme version of a common behavioral paradigm—female philopatry and obligate male migration. These results argue that mitonuclear interactions could have affected key features of macaque societies such as species diversity, female-biased sex ratios, mitonuclear phylogenomic discordance, and sexual dimorphism. More generally, these results evidence links between behavior and genome evolution.

## Introduction

In many organisms, genetic networks consist of components that are encoded by the mitochondria and nuclear genomes, even though these genomic compartments have different modes of inheritance, ploidy, and rates of recombination and mutation. For instance, the crucial processes of oxidative phosphorylation (OXPHOS genes), loading of amino acids on mitochondrial tRNAs (ARS2 genes), the formation of the mitochondrial ribosome (MRP genes), and mitochondrial replication (REP genes) all require collaborative interactions between nuclear-encoded proteins and mitochondrial DNA, RNA, and proteins. Interacting components of these genetic systems therefore must co-evolve in the context distinctive evolutionary dynamics in each compartment (Rand et al. 2004; Burton and Barreto 2012; Hill 2020).

Some forms of sex-specific behavior lead to differences in the spatial distribution of genetic variation in mitochondrial DNA, which is maternally inherited, and autosomal DNA, which is biparentally inherited (Melnick and Hoelzer 1992; Caballero 1995; Hoelzer 1997; Evans and Charlesworth 2013). For example, female philopatry geographically anchors diverged mitochondrial lineages, and if this is coupled with obligate male migration, mitochondrial lineages from philopatric females will be constantly exposed to novel and not co-evolved genetic variation in the autosomes that are imported from afar by migrating males. Added to this, the rate of mutation in mitochondrial DNA is ∼20× faster than in autosomal DNA in many mammals (Osada and Akashi 2012; Allio et al. 2017), and natural selection is expected to be less efficient at purging deleterious mutations from the mitochondrial genome compared with the autosomes because the former is nonrecombining and has a smaller effective population size. Mitonuclear interactions thus are hypothesized to be an example of a Red Queen effect where nuclear genes must constantly evolve to maintain function with the mitochondrial genome; this effect has potentially ancient contributions to fundamental aspects of biology such as the origin of the nucleus, sexual reproduction, genome complexity, germline sequestration, and speciation (Martin and Koonin 2006; Lane 2014; Radzvilavicius et al. 2016; Hill 2017, 2019).

Macaque monkeys are compelling subjects for the study of mitonuclear interactions because females exhibit extreme philopatry and males generally migrate from their natal group (Dittus 1975; Pusey 1987; Swedell 2010; Clutton-Brock and Lukas 2012). These sex differences in migratory behavior lead to sharp differences in mitochondrial diversity between populations, whereas variation in nuclear DNA may be comparatively undifferentiated, even across vast geographic distances (Melnick and Hoelzer 1992; Melnick et al. 1993; Hoelzer 1997; Evans et al. 2001, 2003, 2020). For example, rhesus macaques have the largest distribution of any nonhuman primate (Wolfheim 1983; Southwick et al. 1996) and though as many as 13 subspecies have been previously proposed (Fooden 2000), recent taxonomic assessments consider this species to be morphologically homogeneous (Roos and Zinner 2015). Despite this, there is considerable divergence between intraspecific mitochondrial lineages (Smith and McDonough 2005; Zinner et al. 2013; and see below). Extant rhesus mitochondrial genomes evolved from a most recent common ancestor (MRCA) about 2 Ma (Roos et al. 2019; Evans et al. 2020), which is roughly ten times older than the MRCA of human mitochondria (Fu et al. 2013). Highly diverged mitochondrial genomes are also found in different populations of other widespread macaque species, including pig-tailed (Evans et al. 2020) and long-tailed macaques (Yao et al. 2017), and in other papionin monkeys that share this behavioral system, such as anubis baboons (Roos et al. 2020) and mandrills (Telfer et al. 2003). Additionally, there are striking examples of interspecies introgression of mitochondrial genomes in macaques. Mitochondria of stump-tailed macaques, *Macaca arctoides*, is most closely related to *fascicularis* group macaques (*Macaca mulatta*, *Macaca fascicularis*), whereas the nuclear genome is most closely related to *sinica* group macaques (*Macaca assamensis*, *Macaca thibetana*), suggesting ancient introgression (Tosi et al. 2000; Fan et al. 2018). Likewise, a subspecies of long-tailed macaques, *M. fascicularis aurea*, carries introgressed mitochondria from an ancestor of *sinica* group macaques (Matsudaira et al. 2018)—probably an ancestor of *Macaca sinica* (Evans et al. 2020). These examples illustrate the capacity of the nuclear genome to have compatible interactions with highly diverged mitochondrial genomes.

Recently, an analysis of *silenus* and Sulawesi group macaques recovered signs of natural selection on mitonuclear interactions (Evans et al. 2021). Here, we attempt to generalize these results across other components of macaque diversity by analyzing four genomic data sets: (1) 79 wild *M. mulatta* from China, (2) 89 captive *M. mulatta* from India, (3) *M. arctoides* and close relatives (10 individuals from 5 species), and (4) *M. f. aurea* and close relatives (7 individuals from 3 species, including 2 subspecies of *M. fascicularis*). Our results provide evidence for pervasive and recurrent natural selection on autosomal genes that interact with mitochondrial DNA, RNA, and protein (*N*_interact_ genes), arguing for the possibility that mitonuclear interactions may have influenced the evolution of several distinctive features of macaque societies.

## Results

We used genomic data to perform de novo assemblies of mitochondrial genomes then estimated phylogenetic relationships among the mitochondrial genomes carried by each sample in each of four genomic data sets. Then, for each data set, we performed tests for natural selection on autosomal *N*_interact_ genes, including comparisons between *N*_interact_ and non-*N*_interact_ genes of residence in runs of homozygosity (ROH), population differentiation (*F*_ST_), pairwise nucleotide diversity of polymorphic positions (*π*), Tajima's *D* statistic, and the rate ratio of nonsynonymous to synonymous substitutions per site (d*N*/d*S*). These analyses were focused on groups of individuals defined by phylogenetic relationships among complete mitochondrial genomes and/or taxonomy.

### Mitochondrial Phylogenetics

**Fig. 1.**
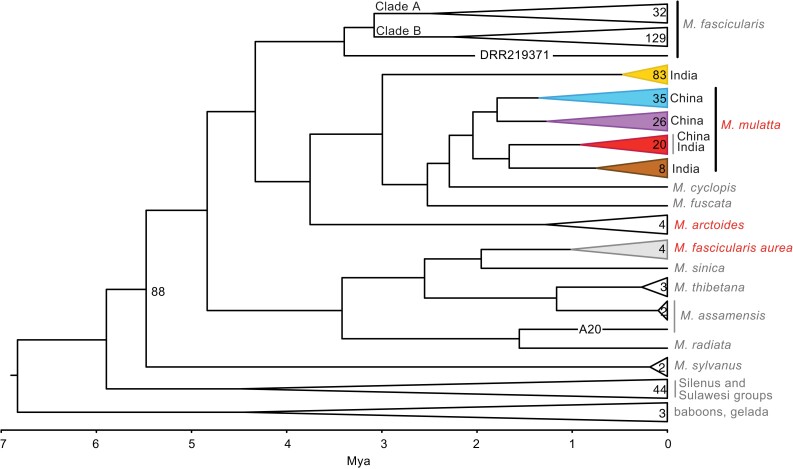
Consensus tree from Bayesian analysis of 402 complete mitochondrial genome sequences from macaques and three outgroup genomes (two baboons, one gelada). Focal species are in red font and the scale bar indicates Ma. All nodes have 100% posterior probability except where labeled with a posterior probability of 88%. Numbers inside collapsed clades refer to the number of genomes, which sometimes are greater than the number of individuals whose autosomal data were analyzed because other mitogenomes are included in this phylogeny (Materials and Methods). A diverged mitochondrial genome from a *Macaca fascicularis* individual from Vietnam is labeled on one branch with its accession number (DRR219371) and a diverged mitochondrial genome from a *M. assamensis* individual is labeled with its sample ID (A20). Colors correspond to the clade designations in the main text.

Inferred evolutionary relationships among major lineages of 405 complete mitochondrial genomes, including 183 that were de novo assembled for this study, are depicted in figure 1. Within *M. mulatta*, three clades were carried by wild macaques from China (hereafter Red, Blue, Purple, with 19, 34, and 26 individuals, respectively, with the Blue clade also including a GenBank genome: KJ567051.1). Two other clades were carried by all but one captive *M. mulatta* from India (hereafter Orange and Brown, with 81 and 8 individuals, respectively, with the Orange clade also including two GenBank genomes: KJ567053.1, AY612638.1). One captive *M. mulatta* putatively from India (MMUL_IN_32754) carried mitochondria from the Red clade. The captive populations of *M. mulatta* from India and three of five subspecies of *M. mulatta* from China (all except *M. m. brevicaudus* and *M. m. tcheliensis*) have paraphyletic relationships among their mitochondrial genomes. Because we are interested in mitonuclear interactions, our analyses of *M. mulatta* are based on inferred mitochondrial clades (Blue, Red, Purple, Orange, and Brown), as opposed to using subspecies taxonomy or captive research centers (see Supplement for more details). Mitochondrial relationships within the focal taxa *M. arctoides* and *M. f. aurea* are monophyletic based on four genomes from each taxon. Other details about mitogenomic phylogenetic relationships are discussed in Supplement.

### Runs of Homozygosity

ROHs are genomic regions that are contiguous genomic tracks where homozygous genotypes are present. The lengths and abundances of ROHs are associated with several phenomena including inbreeding, demographic changes, recombination rates, and natural selection (Ceballos et al. 2018). In humans, for example, ROHs are more commonly observed surrounding alleles that have been subject to natural selection (Pemberton et al. 2012), such as alleles for lactase persistence (Tishkoff et al. 2007), than expected by chance. If *N*_interact_ genes are frequently subject to natural selection, we expected them to more frequently reside in ROH, and for these ROHs to be longer than other ROHs that contain only non-*N*_interact_ genes.

**Fig. 2.**
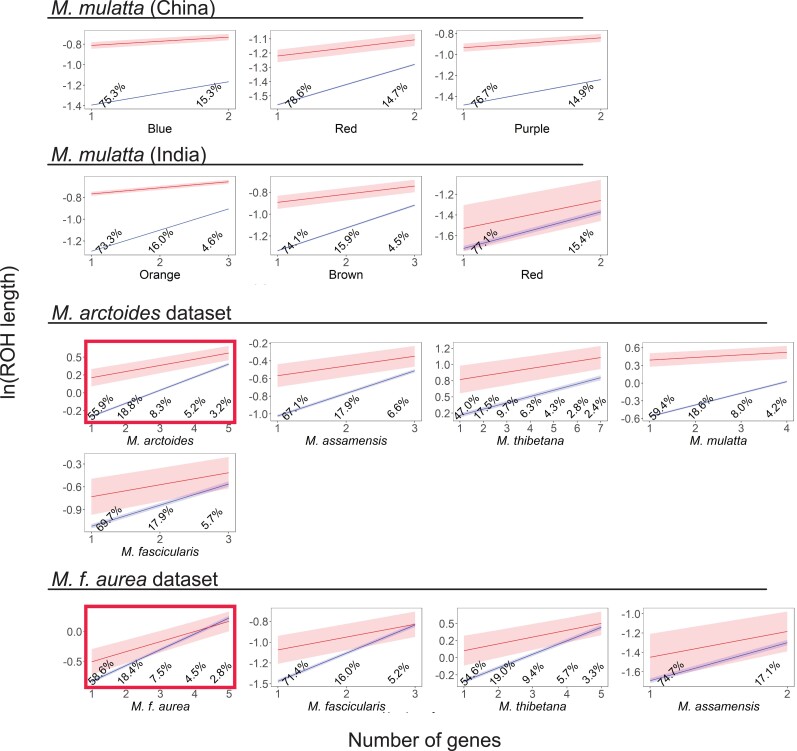
In most species or population in all four data sets, the predicted values (marginal means) of the natural logarithm of the lengths of ROHs (in units of 100 kb) are longer if they contain *N*_interact_ genes (red) compared with those that contain only other genes (blue) across biologically relevant numbers of genes in each ROH (number of genes). The limit of each *x*-axis is the 90th percentile for number of genes in ROHs for each population or species; percentages indicate the proportion of all ROHs that contain each of the plotted numbers of genes. Shading indicates the 95% confidence interval of the predicted values. Red boxes highlight two species or subspecies where we expected a particularly prominent effect of *N*_interact_ genes on ROH length.

We recovered support for this expectation using linear models and nonparametric permutation tests. For all linear models, there was a significant negative interaction between the effect of *N*_interact_ genes and gene number; this indicates that the effect of *N*_interact_ genes on ROH length is smaller or even negative in ROHs that contained many genes (supplementary table S1, Supplementary Material online). One way to evaluate the biological relevance of this interaction is to consider ROHs in terms of the distribution of gene numbers that each ROH carries (from one to the maximum carried by any ROH that has at least one gene). For the ranges of ROH gene numbers from the 1st to the 90th percentiles, the estimated marginal means (predicted values) of all linear models indicated that ROHs that contained *N*_interact_ genes are longer than those that contained only other (non-*N*_interact_) genes (fig. 2, supplementary table S1, Supplementary Material online). These disparities are also evident in density plots which indicate that some ROHs with *N*_interact_ genes are atypically long compared with ROHs with only other genes or ROHs with no genes (supplementary figs. S1–S4, Supplementary Material online). Put another way, for most ROHs that contain biologically relevant gene numbers, ROHs with *N*_interact_ genes tend to be longer than those that carry only other genes.

Permutation tests indicated that *N*_interact_ ROHs were individually significantly longer than non-*N*_interact_ ROHs in only one population (*M. mulatta* in the *M. arctoides* data set; supplementary table S1, Supplementary Material online), but across all comparisons, the permutation tests also indicated that *N*_interact_ ROHs were significantly longer (*P* = 0.020, *χ*^2^ test, degrees of freedom = 30, Fisher's method for combining probabilities). We note that these permutation tests do not control for the interaction between ROH length and gene number but are less affected by autocorrelation than the linear models.

### Genomic Windows: *F*_ST_, *π*, Tajima's *D*

**Fig. 3.**
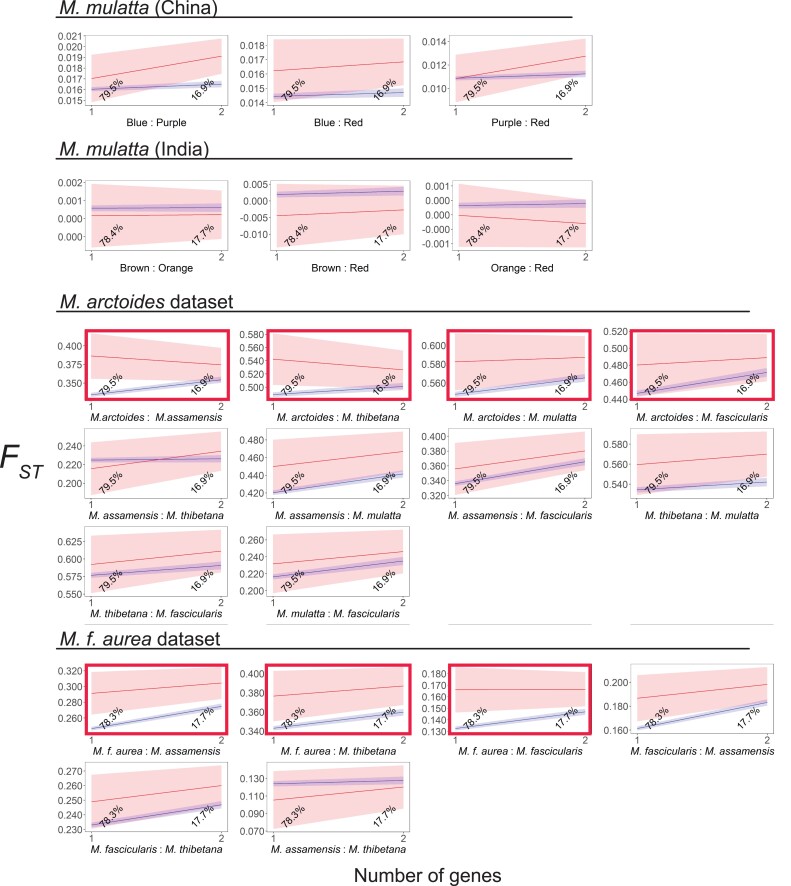
In three of the four data sets (all but the *Macaca mulatta* data set from India), the predicted values (marginal means) for most species or population of *F*_ST_ in 30 kb windows are generally higher in *N*_interact_ windows (red) compared non-*N*_interact_ windows (blue) across biologically relevant numbers of genes in each window (number of genes). Plotting, shading, and highlights follow figure 2 except that here confidence intervals were obtained from block bootstrapping rather than standard errors from linear models.

*F*_ST_ is an index of population subdivision that varies from 0 (no subdivision) to 1 (fixation of different alleles in each population). We expected *F*_ST_ of *N*_interact_ windows (genomic windows that contain the start of transcription of at least one *N*_interact_ gene) to be higher than non-*N*_interact_ windows (genomic windows that contain the start of transcription of at least one non-*N*_interact_ gene but no *N*_interact_ genes) due to species-specific natural selection on mitonuclear interactions. With the exceptions of *M. mulatta* from India and a handful of pairwise comparisons in other data sets, linear models generally found support for this expectation (fig. 3, supplementary fig. S5, tables S2 and S3, Supplementary Material online). Permutation tests also found *F*_ST_ to be significantly higher in *N*_interact_ windows compared with non-*N*_interact_ windows in most individual comparisons (supplementary tables S2 and S3, Supplementary Material online). With the caveat that *F*_ST_ values are not independent across pairwise comparisons within a data set, which is an assumption of Fisher's method for combining probabilities, we note that these comparisons are also significant across all comparisons (*P* ≪0.0001 for both window sizes, *χ*^2^ test, degrees of freedom = 44, Fisher's method for combining probabilities, supplementary tables S2 and S3, Supplementary Material online).

**Fig. 4.**
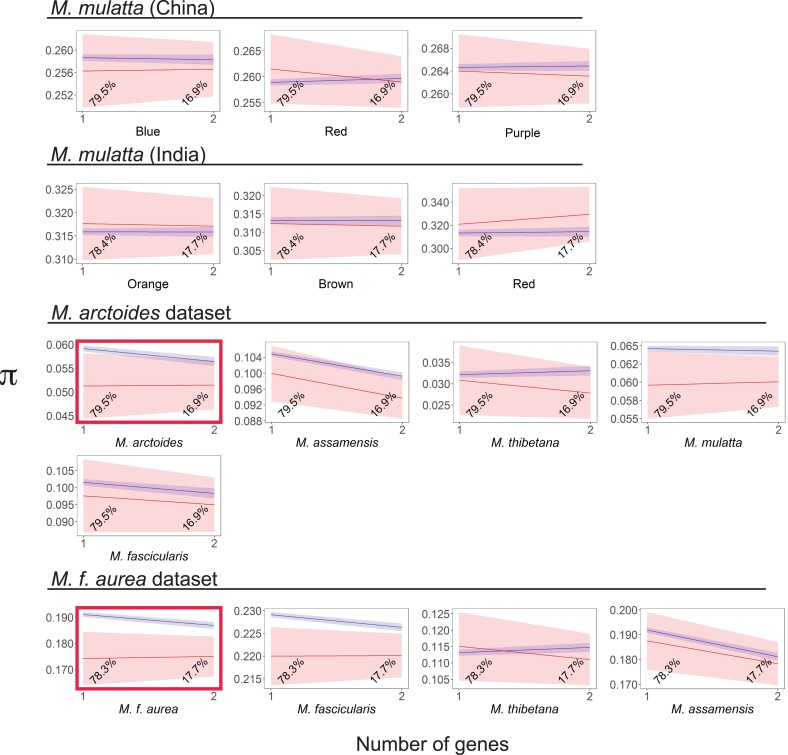
In the *M. arctoides* and *M. f. aurea* data sets but not in *M. mulatta* from China and India data sets, the predicted values (marginal means) for most species or population of *π* in 30 kb windows are generally lower in *N*_interact_ windows (red) compared non-*N*_interact_ windows (blue) across biologically relevant numbers of genes in each window (number of genes). Plotting, shading, and highlights follow figure 3.

Pairwise nucleotide diversity (*π*) is the average proportion of nucleotide differences between all pairs of sequences in a population (Nei 1987). We expected *N*_interact_ windows to have lower *π* than to non-*N*_interact_ windows due to selection on mitonuclear interactions. Consistent with this expectation, the predicted *π* was often lower in *N*_interact_ windows compared with non-*N*_interact_ windows over biologically relevant numbers of genes in each window (fig. 4, supplementary fig. S6, tables S4 and S5, Supplementary Material online). This was particularly apparent in *M. f. aurea* and also in *M. arctoides* but with more variation among window sizes in *M. arctoides* (fig. 4, supplementary fig. S6, Supplementary Material online). Permutation tests also found *π* to be significantly lower in *N*_interact_ windows compared with non-*N*_interact_ windows in several individual comparisons (supplementary tables S4 and S5, Supplementary Material online), and across all comparisons (*P* ≪0.0001 for both window sizes, *χ*^2^ test, degrees of freedom = 30, Fisher's method for combining probabilities, supplementary tables S3 and S4, Supplementary Material online).

Tajima's *D* (Tajima 1989) is a population genetic statistic that is influenced by demography and natural selection; negative values indicate an excess of low frequency polymorphisms, which is a hallmark of expansion in population size or of a recent selective sweep (Fay and Wu 1999). In a constant-sized Wright–Fisher population with no natural selection, the expected value of Tajima's *D* is zero, but demographic changes in natural populations may cause a different genome-wide mean value of Tajima's *D*, even in the absence of natural selection. For this reason, we compared the overall distributions of Tajima's *D* in genomic regions in *N*_interact_ windows, non-*N*_interact_ windows, and nongenic windows, with the expectation that the distribution of Tajima's *D* in *N*_interact_ windows might be more negative or have a more negative tail than the other categories. Because the within species sample sizes were small for the *M. arctoides* and *M. f. aurea* data sets, we restricted our analysis of Tajima's *D* to the two *M. mulatta* data sets. Consistent with our expectation, our findings indicate that Tajima's *D* in *N*_interact_ windows was more negative (or less positive) compared with non-*N*_interact_ windows for four of five (30 kb windows) or five of five (100 kb windows) of the *M. mulatta* populations we tested, and this difference was significant in one of five (30 kb windows) or two of five (100 kb windows) populations according to permutation tests (supplementary table S6, Supplementary Material online) and across all comparisons for both window sizes (*P* < 0.02 for each window size, *χ*^2^ test, degrees of freedom = 10, Fisher's method for combining probabilities).

### Outlier Analysis of *F*_ST_ and *π*

The analyses of genomic windows detailed above were frequently consistent with our expectations associated with natural selection on mitonuclear interactions, but there were several exceptions where the expected difference between *N*_interact_ and non-*N*_interact_ windows was negligible. One possibility is that there could be some *N*_interact_ genes that had atypically high *F*_ST_ and/or low *π* even though the trends for all *N*_interact_ genes were sometimes similar to non-*N*_interact_ genes. This is suggested by density plots that indicate that some *N*_interact_ windows in most data sets have atypically high *F*_ST_ and atypically low *π* compared with non-*N*_interact_ windows (supplementary figs. S7–S14, Supplementary Material online).

To explore this possibility, we performed an outlier analysis with the 30 kb windows to test whether there was an excess of upper *F*_ST_ outliers or lower *π* outliers in *N*_interact_ windows compared with non-*N*_interact_ windows (Materials and Methods and Supplementary Information). This analysis identified an individually significant excess of upper *F*_ST_ outliers in almost all analyses (*P* < 0.05 for all comparisons except Brown:Orange and Brown:Red for the captive *M. mulatta* from India, binomial tests, Supplementary Results, supplementary table S7 and figs. S15–S18, Supplementary Material online). The excess of upper *F*_ST_ outliers were collectively significant across these data sets (*P* ≪0.0001, *χ*^2^ test, degrees of freedom = 44, Fisher's method for combining probabilities). There also was an individually significant excess of lower *π* outliers for all taxa for all data sets (*P* < 0.05, binomial tests, Supplementary Results, supplementary table S8 and figs. S19–S22, Supplementary Material online) that were also collectively significant across all species or populations (*P* ≪0.0001, *χ*^2^ test, degrees of freedom = 30, Fisher's method for combining probabilities).

### The Rate Ratio of Nonsynonymous to Synonymous Substitutions per Site (d*N*/d*S*)

We expected protein evolution to be faster in *N*_interact_ genes compared with non-*N*_interact_ genes. Consistent with this expectation, for all four focal species, the difference between the mean d*N*/d*S* ratio of *N*_interact_ genes and randomly selected non-*N*_interact_ genes was positive, and permutation tests indicate that the observed difference was individually significant for two of four data sets (the *M. arctoides* and *M. f. aurea* data sets; supplementary table S9, Supplementary Material online). The rate of protein evolution was collectively significantly faster in *N*_interact_ compared with non-*N*_interact_ proteins across all data sets (*P* = 0.0001, *χ*^2^ test, degrees of freedom = 8, Fisher's method for combining probabilities).

### Congruent Signals of Natural Selection on the Same *N*_interact_ Genes in Different Species

Comparison of statistics discussed above indicates that they provide both consistent and complementary insights into natural selection. For example, all (*π*) or almost all (*F*_ST_) *N*_interact_ outliers were also found in an *N*_interact_ ROH in at least one individual in the population or species being considered. Likewise, several genes that were upper outliers for *F*_ST_ were also lower outliers for *π* (supplementary table S10, Supplementary Material online). However, *F*_ST_ and *π* outliers were not observed to have higher d*N*/d*S* values compared with non-*N*_interact_ genes (*P* > 0.05, permutation tests), which suggests that these metrics track distinctive signatures of natural selection.

These statistics also provide evidence that several *N*_interact_ genes have been independently subjected to natural selection multiple times in different evolutionary lineages. Specifically, several *N*_interact_ windows were upper *F*_ST_ outliers in multiple comparisons (supplementary figs. S23–S26, Supplementary Material online) or lower *π* outliers in multiple populations or species (supplementary figs. S27–S30, Supplementary Material online). Ten *N*_interact_ windows were upper *F*_ST_ outliers and also were lower *π* outliers in at least four comparisons (*F*_ST_) and populations/species (*π*). These ten windows contain the following *N*_interact_ genes (and functional categories): CCDC56, CD14, NDUFA2, NDUFB8 (OXPHOS); HARS2 (ARS2); MRPL10, MRPL2, MRPL43, MRPL49, MRPL55 (MRP). As discussed in Supplement, many of these genes also were independently identified in a study of mitonuclear interactions in *silenus* group macaques that were not studied here (Evans et al. 2021).

### Analyses of Introgression

Another way that natural selection might influence mitonuclear interactions is by favoring or disfavoring gene flow of regions that contain *N*_interact_ genes, depending on the phylogenetic affinities of the mitochondrial genomes of donor and recipient populations. To explore this possibility, we focused on the *M. arctoides* and *M. f. aurea* data sets because we were able to develop phylogenetically based expectations regarding introgression for each one. For *M. arctoides*, which carries mitochondrial DNA that is closely related to that of *M. mulatta* but whose nuclear genome is more closely related to sinica group macaques (*M. thibetana*, *M. assamensis*), we expected gene flow between *M. mulatta* and *M. arctoides* to be higher in *N*_interact_ windows than in non-*N*_interact_ windows. For *M. f. aurea*, which carries mitochondrial DNA that is closely related to that of sinica group macaques but whose nuclear genome is more closely related to other populations of *M. fascicularis*, we expected gene flow between *M. f. aurea* and *M. assamensis* (or *M. thibetana*) to be higher in *N*_interact_ windows than in non-*N*_interact_ windows.

Using an approach based on a hidden Markov model (Peter 2020), which is expected to be sensitive to recent introgression, we did not detect evidence for extensive recent introgression between *M. arctoides* and the other species in this data set (supplementary figs. S31 and S32, Supplementary Material online). Using this same approach, we also did not detect evidence for extensive recent introgression between *M. f. aurea* and the other species in this data set, including the other *M. fascicularis* genomes (supplementary figs. S33 and S34, Supplementary Material online). Thus, these analyses were uninformative with respect to our predictions. Interestingly and unrelated to our predictions concerning *N*_interact_ genes, we detected evidence of gene flow from *M. arctoides* to one of the *M. assamensis* individuals, an observation that has not been previously reported (e.g., Song et al. 2021).

We also explored our expectations using Patterson's *D* statistic, which potentially could capture signatures of ancient introgression. Consistent with previous inferences, genome-wide introgression patters based on Patterson's *D* statistic indicate that gene flow between *M. mulatta* and *M. arctoides* is greater than between *M. mulatta* and *M. assamensis* or *M. thibetana*; this statistic also indicates that gene flow between *M. f. aurea* and *M. assamensis* or *M. thibetana* is greater than between *M. fascicularis* and *M. assamensis* or *M. thibetana*. However, no significant excess of gene flow was observed for *N*_interact_ windows compared with non-*N*_interact_ windows for any of these comparisons (supplementary table S11, Supplementary Material online). Taken together, these findings fail to provide evidence that recent introgression had a large effect on mitonuclear interactions in *M. arctoides* or *M. f. aurea*, though we note that this does not rule out the possibility of more subtle effects that we were unable to detect.

## Discussion

At least three nonexclusive mechanisms are thought to contribute to selection on mitonuclear interactions: (1) adaptive divergence, where variation in mitonuclear interactions is tuned to unique local conditions, (2) compensatory coadaptation, where changes in the mitochondrial genome lead to selection on *N*_interact_ genes for variants that restore or maintain function, and (3) intergenomic conflict, where the maternal inheritance of mitochondrial DNA prevents efficient purging of deleterious mutations with male-specific effects or when the nuclear genome must evolve coping mechanisms to curtail selfish replication of mitochondrial DNA (Wolff and Gemmell 2013; Chou and Leu 2015; Havird et al. 2019). In general, the relative contributions of adaptive, neutral, and compensatory processes in sculpting mitonuclear interactions are unclear (Wolff et al. 2014), and we consider the combined effects of these factors here.

Across four genomic data sets from macaques, we found that *N*_interact_ genes are embedded in atypically long ROHs, frequently reside in genomic windows with atypically high *F*_ST_ and low *π* and Tajima's *D*, and frequently have atypically rapid rates of protein evolution compared with non-*N*_interact_ genes. After controlling for effects of gene number, every species or population had an individually significant excess of lower *π* outliers in *N*_interact_ windows compared with non-*N*_interact_ windows, and almost every comparison had an individually significant excess of upper *F*_ST_ outliers; across all data sets there was a significant excess of both of these types of outliers. Polymorphism-based metrics of natural selection (ROHs, *F*_ST_, *π*) were frequently concordant, which suggests they are influenced by similar or the same evolutionary processes, though these polymorphism metrics did not strongly overlap with rapid protein evolution (d*N/*d*S*). This disparity could reflect independent selection on regulatory and protein evolution and/or the relatively coarse perspective that our assay of protein evolution provided because the model assumed constraints on protein evolution to be constant across entire proteins and over time.

Signatures of natural selection were frequently concordant in independent comparisons involving different sets of species or populations. This included independent comparisons in this study and also comparisons in the *silenus* and Sulawesi group species and populations (discussed further in Supplement; Evans et al. 2021). Together, these findings argue that mitonuclear interactions of subsets of *N*_interact_ genes from each of the four functional categories (OXPHOS, ARS2, MRP, REP) had a detectable, recurrent, and persistent influence on genome evolution across most or all macaques. This influence likely extends to other papionins that share this social system and perhaps also to other species with similar patterns of sex-biased dispersal.

### How Might Mitonuclear Interactions Influence Macaque Societies?

*Species diversity*. Mitonuclear interactions are proposed to contribute to speciation via epistatic Dobzhansky–Muller interactions between interacting mitochondria-encoded and nuclear-encoded factors (Burton and Barreto 2012; Hill 2017). In macaques, female philopatry and obligate male dispersal causes genetic variation in the autosomes to be more evenly distributed over landscapes than the mitochondria. This could plausibly create conditions that increase the chances of epistatic Dobzhansky–Muller incompatibilities that promote speciation as compared to species where both sexes disperse at similar rates. Indeed, macaques are one of the most diverse and widely distributed genera of Old World monkeys (Roos and Zinner 2015), and there is considerable evidence for natural selection on mitonuclear interactions in macaques (this study; Bailey and Stevison 2021; Evans et al. 2021). This raises the possibility that mitonuclear interactions associated with strong female philopatry increase the rate of speciation. However, some primate groups such as gibbons have less sex-biased migration but are also quite diverse. An interesting direction for future work would test in a phylogenetic context whether the rate of speciation is positively correlated with the extent of male-biased dispersal in mammals, or more specifically in primates.

*Adaptation*. Owing to their central role in energy metabolism, mitonuclear interactions influence essentially all phenotypes of eukaryotes (Rand and Mossman 2020). Macaques inhabit extraordinarily diverse habitats spanning rain forests of Southeast Asia, savanna and scrublands of India, mountainous regions of Morocco, Pakistan, Nepal, and China, areas with seasonal snow in the mountains of Japan, and even urban habitats. They are generally frugivorous, but their flexible diet also may include invertebrates, plants, and meat (Holzner et al. 2019). Might adaptive mitonuclear epistasis account for some component of the ecological breadth of macaque monkeys, wherein certain combinations of mitochondrial and nuclear variation are beneficial in certain contexts? One way to empirically test this is with xenomitochondrial hybrids (Kenyon and Moraes 1997; McKenzie et al. 2003), which could ethically be accomplished using cell culture in macaques. In fruit flies, this approach identified context-dependent tradeoffs that are associated with mitonuclear interactions (Camus et al. 2020), thereby demonstrating that natural selection can favor different mitochondrial variation in different environments, even while holding variation in the nuclear genome constant.

*Discordance between mitochondrial and nuclear phylogenies*. Many examples exist in macaques where relationships among mitochondrial DNA do not match those among nuclear DNA (mitonuclear phylogenomic discordance; see Supplement for examples). As discussed above, differences in male and female dispersal contribute to mitonuclear phylogenomic discordance, and compensatory evolution in the nuclear genome may contribute as well. Another nonexclusive possible contributor to mitonuclear phylogenomic discordance is if the deleterious load on the mitochondrial genome in one population creates opportunity for invasion of a less-loaded mitochondrial genome from a neighboring population or species (Sloan et al. 2017). Added to this, epistatic incompatibilities need not be symmetrical in the sense that a cross between a female from one group and a male from another may produce fit offspring, but not vice versa (Darwin's corollary to Haldane's Rule; Turelli and Moyle 2007). Together these mitogenomic factors could contribute to dissimilar patterns of introgression of mitochondrial and nuclear variation in macaques, thereby increasing mitonuclear phylogenomic discordance.

*Female-biased adult sex ratio*. Macaques often have highly skewed female-biased sex ratios (Lindburg 1969; Van Schaik et al. 1989; Beisner et al. 2012) and this is also the case in other papionins (Swedell 2010). One possible contribution to this skew is that deleterious effects associated with mitonuclear incompatibilities disproportionately target males because natural selection is inefficient at removing deleterious mitochondrial mutations that have male-specific or male-biased effects (“mother’s curse”; Gemmell et al. 2004; Wolff and Gemmell 2013; Gemmell 2015). This possibility is supported in human by associations between mitochondrial variation and male infertility (Frank and Hurst 1996; Gemmell et al. 2004; Frank 2012). The effects of mother's curse may be mitigated by positive assortative mating by creating circumstances that allow for selection against mitochondrial variation that is only detrimental in males (Hedrick 2012). However, migration of males from their natal groups brings them into contact with females carrying diverged mitochondria; this creates conditions that more closely match negative assortative mating, which has the opposite effect of increasing mother's curse (Hedrick 2012).

*Sexual dimorphism*. Macaque species vary extensively in secondary sexually dimorphic characters such as body size, canine length, and pelage (Nunn 1999; Plavcan 2001). Although not generally considered a component of sexual dimorphism, it is notable that there is considerable between species diversity in genital morphology and estrus swelling among macaques (Fooden 1976, 1989). The genitals of *M. arctoides* are particularly distinguished from other macaques, with males having the largest glans and baculum, and females having distinctions in internal and external morphology of the perineum, cervix, vagina, and position of the urethra (Fooden 1967). Is it a coincidence that the macaque species with the most apomorphic genital morphology also has introgressed mitochondria? An interesting possibility is that intraspecific and interspecific variation in sexual dimorphism could signal mitonuclear compatibility (Hill and Johnson 2013; Hill 2019), or that the expression of sexually dimorphic characteristics signals capacity of the nuclear genome to control selfish mitochondrial replication (Havird et al. 2019). Macaques have intraspecific variation in sexually dimorphic characteristics (Higham et al. 2012; Petersdorf et al. 2017; Rosenfield et al. 2019), but whether and to what degree these features vary with condition (or specifically the efficacy of cellular respiration) is unclear. In fruit flies, variation in mitochondrial genomes has a sex-specific trans-acting effect on expression of nuclear genes (Mossman et al. 2016), demonstrating that mitochondrial variation can have sex-specific effects on the nuclear genome.

*Sex-biased dispersal*. Dispersal can be a risky undertaking because it exposes individuals to unfamiliar habitats that may increase food insecurity, predation, intraspecific competition, and poses challenges to finding a mate (Bonte et al. 2012), but carries benefits associated with outbreeding, decreasing conspecific density and competition among kin, and location of new resources (Hamilton and May 1977; Matthysen 2005). Our efforts focused on macaques because they have an extreme female philopatry that we expected to drive natural selection on mitonuclear interactions. But it is also possible that sex-biased dispersal is an evolutionary response to mitonuclear interactions or (perhaps even more likely) there could be evolutionary feedback between these phenomena. Scrutiny of mitonuclear interactions in *Papio papio* and *Papio hamadryas*, which secondarily lost female philopatry (Kopp et al. 2015; Staedele et al. 2015), and comparison with other papionins may provide further insights into these evolutionary dynamics.

## Conclusions

Our analyses of the most widely distributed nonhuman primate—*M. mulatta—*and two macaques with introgressed mitochondrial genomes—*M. arctoides* and *M. f. aurea—*identifies signatures of natural selection in polymorphism and protein evolution of *N*_interact_ genes. These findings, coupled with theory and findings from other species, argue that mitonuclear interactions potentially have implications for speciation, adaptation, phylogenomics, demography, and sexual selection in macaques and other species with similar patterns of sex-biased dispersal.

## Materials and Methods

### Data

This study considered genomic data from four data sets: (1) 79 wild-sampled *M. mulatta* individuals from China (Liu et al. 2018), (2) captive *M. mulatta* from India (subsetted from 850 to 89 individuals), (3) *M. arctoides* and close relatives (subsetted from 13 to 10 individuals total to exclude three distantly related *silenus* group species), and (4) *M. f. aurea* and close relatives (7 individuals). The first three data sets were previously published, and analysis was performed on genotype (vcf) files from those studies (Liu et al. 2018; Warren et al. 2020; Bailey and Stevison 2021). Additional information on these data is provided in Supplement.

### Mitochondrial Genome Assembly and Phylogeny

As detailed above, we used a mitochondrial phylogeny to guide our analyses of autosomal genes. *De novo* assembly of mitochondrial genomes was performed as described in Supplement. We combined *de novo* assemblies of 183 complete mitochondrial genomes with 222 complete genomes that were obtained from GenBank as detailed in Evans et al. (2020) for a total of 405 complete genome sequences including three outgroup genomes (2 baboons, 1 gelada). Evolutionary relationships among the mitochondrial genomes were estimated using Bayesian and maximum likelihood approaches as detailed in Supplement. *De novo* assemblies of complete mitochondrial genomes are deposited in GenBank (accession numbers OQ199566–OQ199748).

### *N*_interact_ Genes

Using annotation files of two reference genomes from rhesus macaques (versions 8 and 10), we searched for autosomal genes that are involved with any of the four mitonuclear interactions discussed above (OXPHOS, ARS2, MRP, REP; Gaspari et al. 2004; Sissler et al. 2017; Bogenhagen et al. 2018; Signes and Fernandez-Vizarra 2018) and that have direct or very close interactions with mitochondrial DNA, RNA, or protein. We identified 199 autosomal *N*_interact_ genes in each version in the following *N*_interact_ categories: OXPHOS (101 and 100 in versions 8 and 10), ARS2 (77 and 78), MRP (17 in both assembly versions), and REP genes (4 in both versions). OXPHOS complex II was excluded from the *N*_interact_ category, because it comprises only autosomal encoded proteins. A total of 21,094 and 20,897 autosomal non-*N*_interact_ genes were identified in versions 8 and 10, respectively. In both assembly versions, two and zero *N*_interact_ genes were identified on the X and Y chromosomes, respectively. Because several factors influence genetic variation on the sex chromosomes in ways that differ from the autosomes (mutation rate, effective population size, hemizygosity, natural selection), the sex chromosomes were excluded from all analyses.

### Analysis of Polymorphism Within and Between Lineages

We predicted that natural selection on *N*_interact_ genes would decrease variation in these genes and their flanking regions within lineages and increase differentiation in pairwise comparisons between lineages. To test this, we analyzed ROHs and pairwise nucleotide diversity of polymorphic sites (*π*) in groups of individuals defined by mitochondrial variation and taxonomy, and population differentiation (*F*_ST_) between these groups.

ROHs were identified using bcftools version 1.4 (Narasimhan et al. 2016). We defined *N*_interact_ ROHs to be ROHs that contained the start of transcription of at least one *N*_interact_ gene, and non-*N*_interact_ ROHs to be ROHs that contained the start of transcription of at least one non-*N*_interact_ gene but no *N*_interact_ genes. We used linear models to test the hypothesis that the predictor variable *N*_interact_, which indicated whether (1) or not (0), an ROH was an *N*_interact_ ROH, was positively correlated with the natural logarithm of *ROH*_*length* in 100 kb pairs. We log transformed the response variable because the distribution of the untransformed variable was skewed. An interaction term with the number of genes in each ROH (*gene_number*) was included in the linear models for each species or population in each data set. Although we did not have an a priori prediction for the interaction term in this model or in other linear models discussed below, we included this term as a way of avoiding making the strong assumption that our predictor variables were independent. ROHs lacking a start of transcription sites for at least one gene were excluded from this analysis.

*F*_ST_ and *π* were analyzed in genomic windows, and we defined *N*_interact_ windows to be any genomic window that contained the start of transcription of at least one *N*_interact_ gene, and non-*N*_interact_ windows to be any genomic window that contained start of transcription of non-*N*_interact_ genes but no *N*_interact_ genes. We considered two window sizes (30 and 100 kb) and used linear models to test the hypothesis that *F*_ST_ and π were higher or lower, respectively, in *N*_interact_ windows compared with non-*N*_interact_ windows. We included an interaction term between *gene_number*, which is the number of genes in a genomic window, and the predictor variable *N*_interact_, which indicated whether (1) or not (0) a window was an *N*_interact_ window, on the response variable (*F*_ST_ or *π*). Because their distributions were not especially skewed, these response variables were not transformed. *F_ST_* was calculated using the method of Hudson et al. (1992) with a minimum allele frequency cutoff of 0.05; *F*_ST_ of genomic windows was calculate as the average of the *F*_ST_ values at each variable position.

Linked genomic regions tend to have similar genomic properties, which violates the assumption that the response variable of linear models is independently distributed. For this reason, we used block bootstrapping to estimate standard errors of the coefficients of our linear models of genomic windows using the block.glm function of the R package genomic.autocorr version 1.0-1 (Wallace and Burren 2017). Autocorrelation plots that were generated using the acf function of the R stats package version 3.6.2 (R_Core_Team 2018) suggested that autocorrelation decayed within a block size of 30; this value was used for the block bootstrapping for 30 and 100 kb windows with 1,000 replicates. *P*-values were then calculated from standard errors following (Altman and Bland 2011).

Because ROHs were not divided into equally sized windows, we used a combination of linear models and nonparametric permutation tests to evaluate whether the observed difference between *N*_interact_ ROHs length and non-*N*_interact_ ROHs length was significantly higher than expected by chance. In the permutations, the observed difference between the mean ROH length of *N*_interact_ and non-*N*_interact_ ROHs was compared with a distribution of differences calculated from by randomizing the assignment of *N*_interact_ and non-*N*_interact_ ROHs for 1,000 randomizations. We had a one-sided expectation that observed differences should be atypically large compared with the randomized values. As a complement to the block bootstrap analyses discussed above, we also performed these permutations on the genomic window metrics (*F*_ST_, *π*).

Outlier analyses were performed for *F*_ST_ and *π* using Cook's distance, using linear models as in (Evans et al. 2021). This analysis evaluates whether a subset of the data (here *N*_interact_ windows) has more extreme outliers (upper outliers for *F*_ST_, lower outliers for *π*), than expected based on the rest of the data (the non-*N*_interact_ windows). In all analyses, genomic windows lacking a start of transcription sites for at least one gene were excluded. For this analysis, outliers are identified from the entire set of genomic windows; we then evaluate whether there is an excess of outliers that are *N*_interact_ windows.

Tajima's *D* was calculated in 30 and 100 kb genomic windows using vcftools version 0.1.16 (Danecek et al. 2011). Permutation tests with 1,000 replications were used to test the hypothesis that the difference between the mean Tajima's *D* of *N*_interact_ windows and non-*N*_interact_ windows was more negative than expected by chance as described in Evans et al. (2021). This analysis was performed only on the two *M. mulatta* data sets, because the intraspecific sample sizes were small for the *M. arctoides* and the *M. f. aurea* data sets.

### d*N*/d*S*

We expected protein evolution to be faster in *N*_interact_ compared with non-*N*_interact_ genes. To test this, for each data set, a phylogeny was obtained among individuals by first converting the genotypes to nexus files using the vcf2philip script (https://github.com/edgardomortiz/vcf2phylip; Ortiz 2019) and requiring at least 80% of the taxa to have called genotypes. For the *M. arctoides* and *M. f. aurea* data sets, variable positions within the complete genome were used; for the much larger data sets from *M. mulatta* variable positions only on chromosome 1 were used. A phenogram using the neighbor-joining algorithm using PAUP* (Swofford 2002) and this topology was used for analysis of d*N*/d*S*. For each gene, we used the codeml program within the PAML package version 4.9 (Yang 2007) to estimate the rate ratio of nonsynonymous to synonymous substitutions per site (d*N*/d*S*) over each phylogeny under a model with one d*N*/d*S* parameter. This analysis was performed for each *N*_interact_ gene and 3,000 randomly selected non-*N*_interact_ genes. We discarded genes that lacked an “ATG” start site, contained premature stop codons, whose coding region was not a multiple of three nucleotides long, or if the estimated d*N*/d*S* value was >5. Permutation tests were used to test whether the observed difference between the mean d*N*/d*S* ratios of *N*_interact_ and non-*N*_interact_ genes was significantly higher than expected by chance as described above.

### Comparisons Across Data Sets

To assess whether the signatures of natural selection were shared across metrics (*π*, *F*_ST_, ROHs, d*N*/d*S*) and to explore the possibility that the genes are repeatedly subject to natural selection in different species, we identified and quantified genomic windows that were lower *π* outliers and also upper *F*_ST_ outliers using the R package UpSetR (Conway et al. 2017), and we also quantified residence of these outliers in ROHs. In addition, we used permutation tests similar to those detailed above to test whether these outliers also tended to have higher d*N*/d*S* values compared with the non-*N*_interact_ genes.

### Analyses of Introgression

We used two approaches to test for evidence of introgression and if detected, to permit us to evaluate hypotheses regarding introgression of *N*_interact_ and non-*N*_interact_ windows that are articulated in the results. The first approach uses a hidden Markov model to infer introgression in 100 kb windows as implemented by the software admixfrog (Peter 2020). Because the data produced errors under some single-nucleotide polymorphism densities, for each chromosome, we thinned the data to differing degrees, removing variant sites that were within 200–2,000 positions from other variable sites. Because the extent of introgression that was detected in *M. arctoides* and *M. f. aurea* was low, no statistical analyses were performed on these introgression blocks.

The second approach we used to study introgression used Patterson's *D* statistic (Patterson et al. 2012). This analysis was performed using 30 and 100 kb windows and using taxon settings that matched established evolutionary relationships among nuclear genes or genomes (Fan et al. 2018; Matsudaira et al. 2018), namely for the *M. f. aurea* data set (((*M. fascicularis*, *M. f. aurea*), *M. assamensis*), *Macaca nemestrina*) and for the *M. arctoides* data set (((*M. arctoides*, *M. assamensis*), *M. mulatta*), *M. nemestrina*). For both data sets, we also analyzed Patterson's *D* after substituting the *M. thibetana* genome for the *M. assamensis* genomes. For the *M. f. aurea* data set, we used previously published genomic data from an *M. nemestrina* individual (PM1206) as an outgroup; genotypes for this individual were called along with the other individuals in this data set for this analysis only using the pipeline described above. Following Evans et al. (2021), permutation tests were used to evaluate expectations based on evolutionary relationships among mitochondrial genomes of these samples that are described in the results.

## Supplementary Material

evad010_Supplementary_Data

## Data Accessibility

Data from this study were either downloaded from NCBI (accession numbers DRS139837, DRS139838, DRR219371, SRA023855, SRR1564766, SRR2981114, SRR1024051), provided by the authors of (Warren et al. 2020), or obtained from Supplementary Information (Liu et al. 2018; Bailey and Stevison 2021).
