# Supplementary material for: Mitonuclear Interactions and the Origin of Macaque Societies
Source: Genome Biol Evol. 2023 Feb 9;15(2):evad010. doi: 10.1093/gbe/evad010 (PMC9937042; doi:10.1093/gbe/evad010)
Supplement: evad010_Supplementary_Data [file evad010_supplementary_data.pdf]

Supplementary Introduction

Macaque dispersal. In mammals, dispersal is often sex-biased, with the drivers of sex-specific dispersal thought to be influenced by factors such as social system and spatiotemporal variation in sex-specific resources (Hamilton and May 1977; Li and Kokko 2019). Many mammals have male-biased dispersal (Greenwood 1980; Dobson 1982) but with less male-bias than most papionin monkeys (Thierry 2007; Clutton-Brock and Lukas 2012). The extreme male-biased dispersal in most macaque monkeys is due to unusually strong female philopatry (as opposed to unusually long-distance male dispersal) because females generally stay in their natal group for their entire life (Dittus 1975; Pusey 1987; Swedell 2010; Clutton-Brock and Lukas 2012; Fischer et al. 2019).

Supplementary Methods

Data. For the captive rhesus from India, we selected a subset of nine individuals randomly from each of eight captive colonies; for a ninth colony (WNPRC) we analyzed 18 individuals including nine randomly selected individuals from each of two batches of samples from this center, as detailed in (Warren et al. 2020). For one captive rhesus from India, a mitochondrial genome was assembled from raw reads, but genotypic data were lacking, leaving a total of 89 individual captive rhesus from India for which autosomal data was analyzed. For the *M. arctoides* dataset, we excluded data from three individuals from the *silenus* species group to focus our findings on the more closely related species and so that our results would be evolutionarily independent from (Evans et al. 2021). This left ten individuals in the *M. arctoides* dataset, including three *M. arctoides* (SRS6488501, SRS1196878 (SRR2981139), SRS1196879 (SRR2981140) – provenances not reported), two *M. assamensis* (SRS6491954, SRR2981114– provenance not reported), one *M. thibetana* (SRR1024051 – Sichuan, China), one *M. fascicularis* (SRS117874 - Vietnam), and three *M. mulatta* (SRS115022 – SW China, SRS212016 – SW China, SRS114988 – provenance not reported) individuals. For the *M. f. aurea* dataset, genomic data from two *M. f. aurea* individuals (DRS139837, DRS139838) were compared to genomic data from three *M. f. fascicularis* individuals (DRR219371, SRA023855, SRR1564766, from Thailand, Vietnam, and Mauritius respectively), one *M. assamensis* (SRR2981114) and one *M. thibetana* (SRR1024051) individual. For this dataset, data were trimmed, aligned to version 10 of the rhesus genome, de-duplicated, genotyped and filtered as described elsewhere (Evans et al. 2021).

$N_{interact}$ genes. More than 500 nuclear-encoded proteins are imported into the mitochondrial organelle (Mootha et al. 2003; Sickmann et al. 2003) that participate in processes discussed in the main text (OXPHOS, ARS2, MRP, REP) but also a spectrum of other functions, e.g. mitochondrial membrane biogenesis and maintenance, nucleotide synthesis and transport, cytosolic chaperones, mitochondrial mRNA degradation and apoptosis (Smits et al. 2010). We focused here on a subset of these proteins that directly interact with mitochondrial DNA, RNA, or protein because we expected the strongest signature of natural selection on mitonuclear interactions involving these proteins. OXPHOS complex II genes were not considered to be $N_{interact}$ genes because this complex does not contain mitochondria-encoded proteins. Only two $N_{interact}$ genes occur on the sex chromosomes (both are OXPHOS genes on the X chromosome) and genes on the sex chromosomes were excluded from analysis (see main text).

Mitochondrial phylogenomics. *De novo* assembly of mitochondrial genomes was performed using NOVOplasty version 4.3.1 (Dierckxsens et al. 2017). Many genomes were successfully assembled using a kmer size of 29, but we also attempted higher (33, 38) and lower (24) values if the initial assembly was unsuccessful. We included one sample from the captive rhesus from India (MMUL_IN_32510) where we assembled a mitochondrial genome even though there was no genotype data available; this individual was included in the phylogenetic analysis of the mitochondrial genomes but not in the population genetic and molecular evolutionary analyses of autosomal data. For four captive rhesus individuals from India (MMUL.IN-39345, MMUL.IN-38591, MMUL.IN-32510, MMUL.IN-35091), assembly of complete mitochondrial genomes was only achieved after we first mapped raw data to a mitochondrial reference genome (KJ567053.1, a *M. mulatta* from India) and the performed *de novo* assembly using NOVOplasty with only the mapped reads. We were unable to assemble the mitochondrial genome from one *M. fascicularis* individual (SRR1564766, from Mauritius) and we instead assumed that the phylogenetic placement of this individual's mitochondrial genome was the same as a GenBank accession of another longtail macaque from Mauritius (KM851000.1). Similarly, because raw data from the *M. arctoides* dataset were not available at the time of analysis, we included publicly available mitochondrial genomes from each species and geographic region represented in this dataset instead of *de novo* assembled genomes.

Evolutionary relationships among the mitochondrial genomes were estimated using Bayesian and maximum likelihood approaches using the software BEAST version 2.6.3 (Bouckaert et al. 2019) and Iqtree version 1.6.12 (Nguyen et al. 2015) respectively. For the BEAST analysis, the model of evolution, calibration times, and priors were the same as a previous study of mitogenomics (Evans et al. 2020). Ten independent runs were performed, each with a chain length of 43.3–73.2 million generations and sampling parameters every 1000 generations; a burn-in of 50% of each chain was discarded. Tracer was used to evaluate the effective sample sizes of each parameter after discarding the burn-in; all values were greater than 100 and most were greater than 200. The Logcombiner program from the BEAST package was used to combine the post-burn-in posterior distribution of trees and further thin the trees to sample every 5000 generations. Then the Treeannotator program from the BEAST package was used to generate a maximum clade credibility tree. For the maximum likelihood analysis, the TIM2+F+I+G4 model was used based on the Bayesian Information Criterion as implemented by Iqtree; confidence of nodes was evaluated using the ultrafast bootstrap approach (Minh et al. 2013). For both analyses, we excluded a portion of the alignment where the assembly of five wild rhesus samples (C_rhe_28, 29, 30, 32, and 34) had an insertion with homology to the COX2 gene because we were unsure of the validity of this portion of the alignment. Because major topographical relationships were identical in both analyses, we present only the Bayesian consensus tree.

Supplementary Results

Mitochondrial phylogeny

The estimated mitochondrial phylogeny is consistent with previous inferences (Roos et al. 2019) that the mitochondrial genome of *M. arctoides* is most closely related to a clade containing mitochondrial lineages carried by *M. mulatta*, *M. cyclopis*, and *M. fuscata*. The phylogeny also

supports previous inferences (Matsudaira et al. 2018) that there was an ancient introgression of mitochondrial genomes to an ancestor of *M. f. aurea* from an ancestor of *M. assamensis* and *M. thibetana*. As previously (Evans et al. 2020), the inclusion of data from *M. sinica* indicates that this introgression event occurred between an ancestor of *M. sinica* after divergence from the ancestor of *M. assamensis* and *M. thibetana.*

For the captive rhesus macaques, animals that carry the orange clade are derived from ten institutes; sample sizes follow institutional abbreviation from (Warren et al. 2020): (JC_UoC: 6, JH_CPRC: 9, EV_NEPRC: 9, SKDS_CNPRC: 9, ZJ_YNPRC: 6, LPVGTI_OHCU: 9, RWDO_WNPRC: 9, MK_TNPRC: 9, BF_ONPRC: 8, RWNK_WNPRC: 6); animals that carry the brown clade are derived from four institutes (JC_UoC: 3, ZJ_YNPRC: 3, BF_ONPRC: 1, RWNK_WNPRC: 1), and the one individual that carried the red clade came from RWNK_WNPRC.

An unexpected inference came from the mitochondrial genome of a *M. fascicularis* individual from Vietnam (DRR219371) from the *M. f. aurea* dataset, which was highly diverged from the other two widespread mitochondrial lineages in this species (Fig. 1). To our knowledge this diverged mitochondrial lineage has not been previously reported. Also unexpected was the nested position of the *M. sylvanus* lineage relative to the *silenus* group. However, this relationship had lower support in the Bayesian (Fig. 1) and maximum likelihood analysis (bootstrap support of 90%) compared to nodes subtending other major lineages (all had posterior probability and bootstrap support of 100%). Most other analyses suggest that mitochondrial DNA from *M. sylvanus* is sister to that of all other extant macaque species (Roos et al. 2019; Evans et al. 2020).

***ROH* analyses**

For each population or species in each dataset, portions of density plots of ROHs with $N_{interact}$ genes are right shifted compared to ROHs with only other genes and compared to ROHs with no genes (Figs. S1–4).

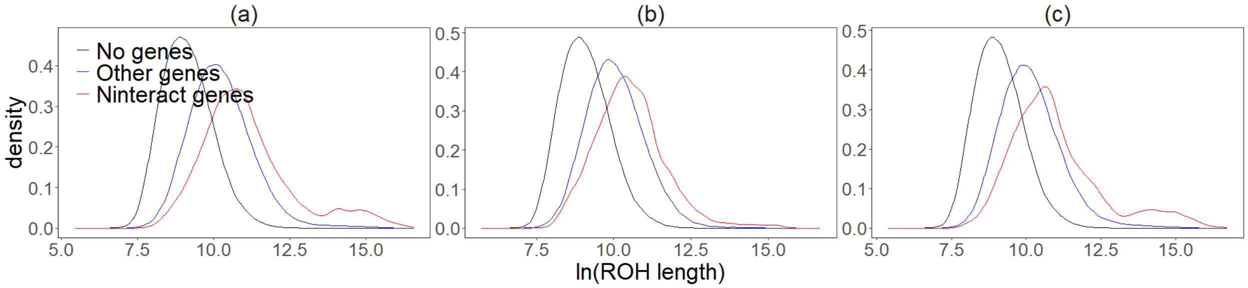

Fig. S1. Density plot of ROHs of wild *M. mulatta* from China illustrates longer length of $N_{interact}$ ROHs (red) compared to ROHs that contain only other genes (blue) or no genes (black). The order of panels matches Fig. S1.

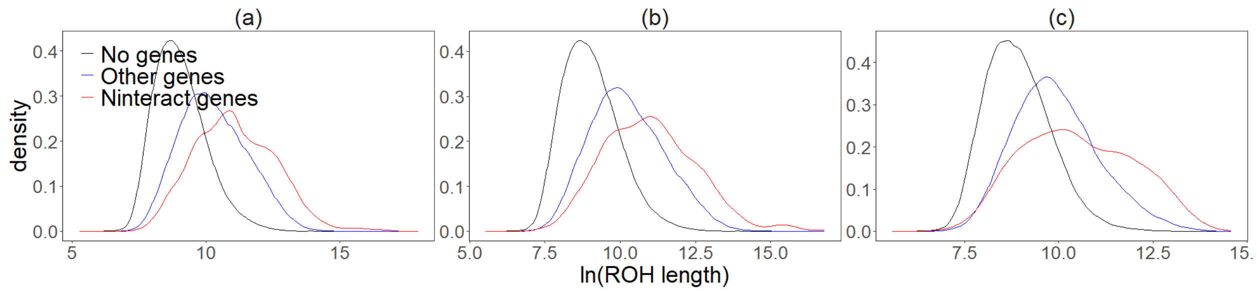

Fig. S2. Density plot of ROHs of captive *M. mulatta* from India illustrates longer length of $N_{interact}$ ROHs (red) compared to ROHs that contain only other genes (blue) or no genes (black). The order of panels matches Fig. S2.

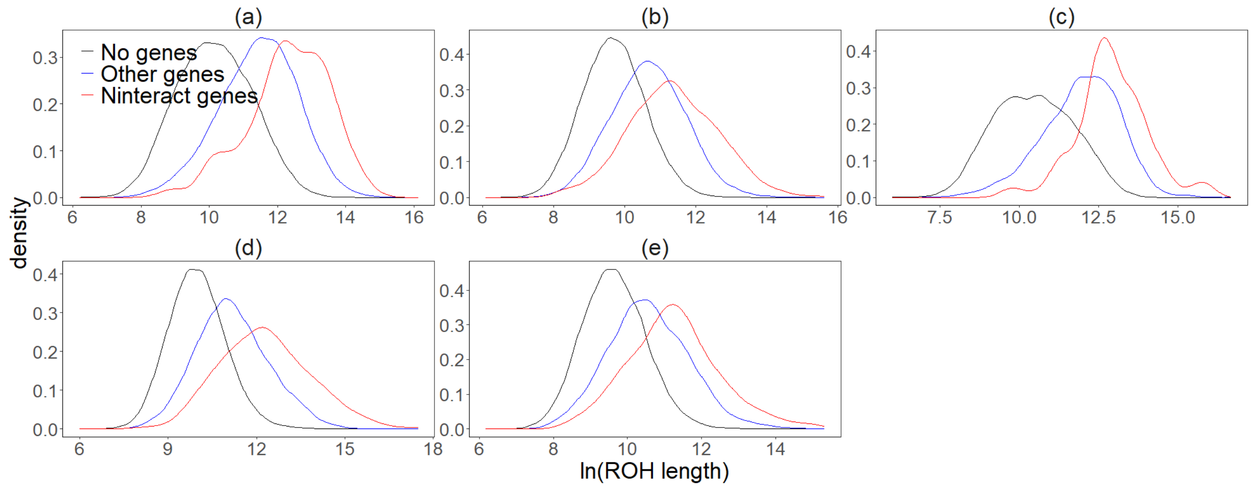

Fig. S3. Density plot of ROHs of *M. arctoides* dataset illustrates longer length of $N_{interact}$ ROHs (red) compared to ROHs that contain only other genes (blue) or no genes (black). The order of panels matches Fig. S3.

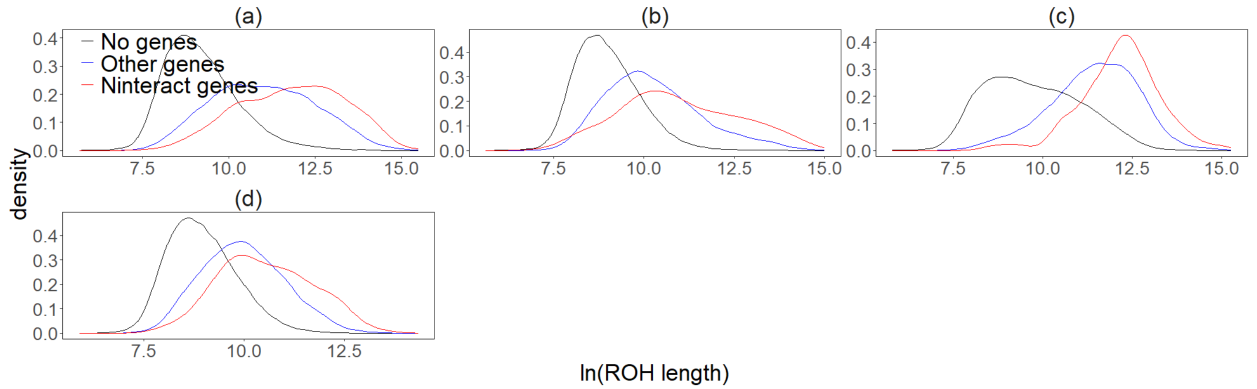

Fig. S4. Density plot of ROHs of *M. f. aurea* dataset illustrates longer length of $N_{interact}$ ROHs (red) compared to ROHs that contain only other genes (blue) or no genes (black). The order of panels matches Fig. S4.

F_{ST} and π in 100kb windows

With exceptions discussed in the main text, linear models fitted to data from 100kb windows (Figs. S5, S6) recovered similar trends as those fitted to data from 30kb windows (Figs. 3, 4) in that F_{ST} predicted by the linear fit was generally higher and π predicted by the linear fit was generally lower in $N_{interact}$ compared to non-$N_{interact}$ windows. Consistent with this, portions of density plots of F_{ST} with $N_{interact}$ windows are right shifted compared to non-$N_{interact}$ windows and compared to windows with no genes (Figs. S7–14).

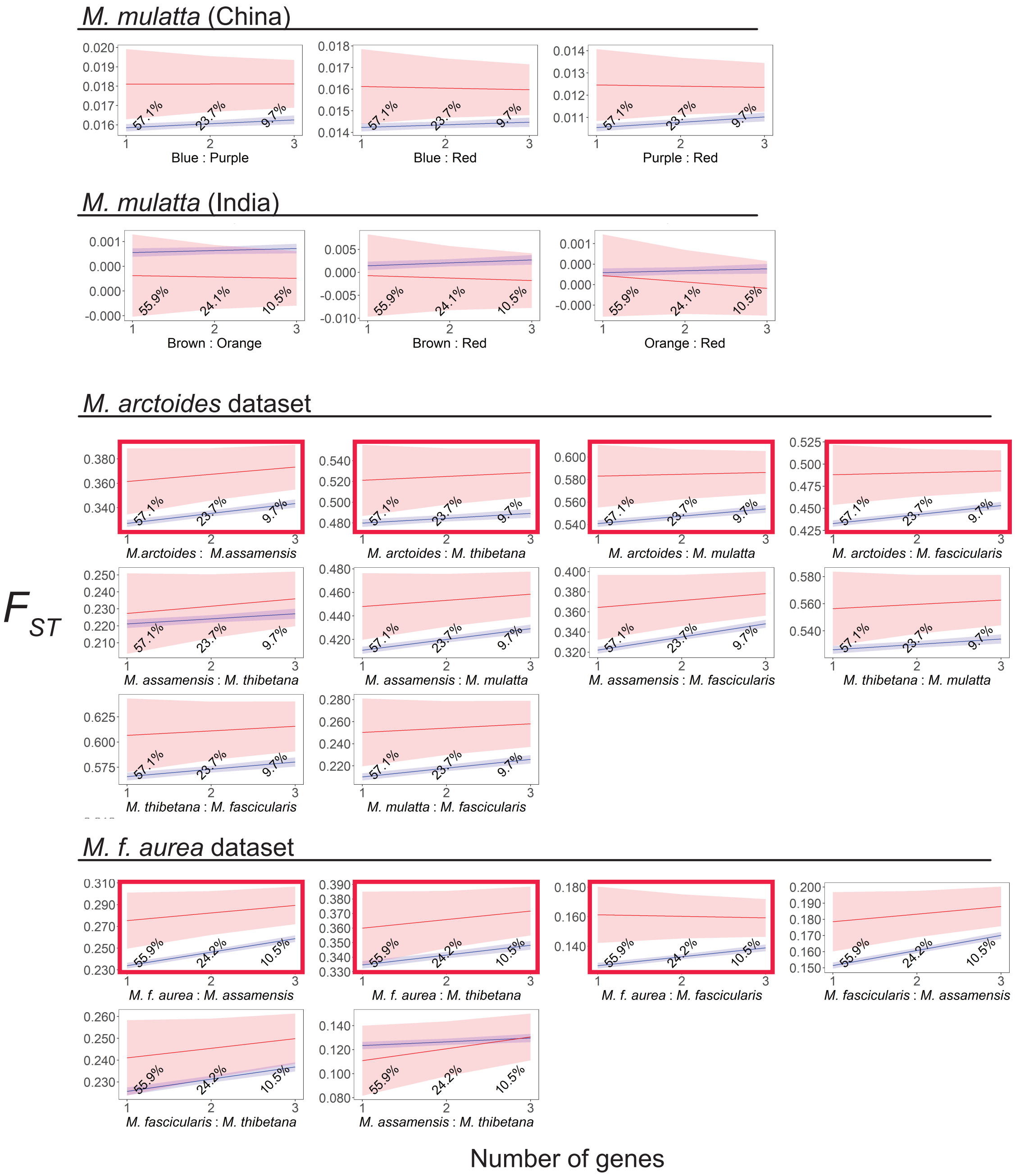

Fig. S5. In three of the four datasets (all but *M. mulatta* from India), the predicted values (marginal means) for most species or population of F_{ST} in 100 kb windows are generally higher in $N_{interact}$ windows (red) compared non-$N_{interact}$ windows (blue) across biologically relevant numbers of genes in each window (Number of genes). Plotting, shading, and highlights follow Fig. 3.

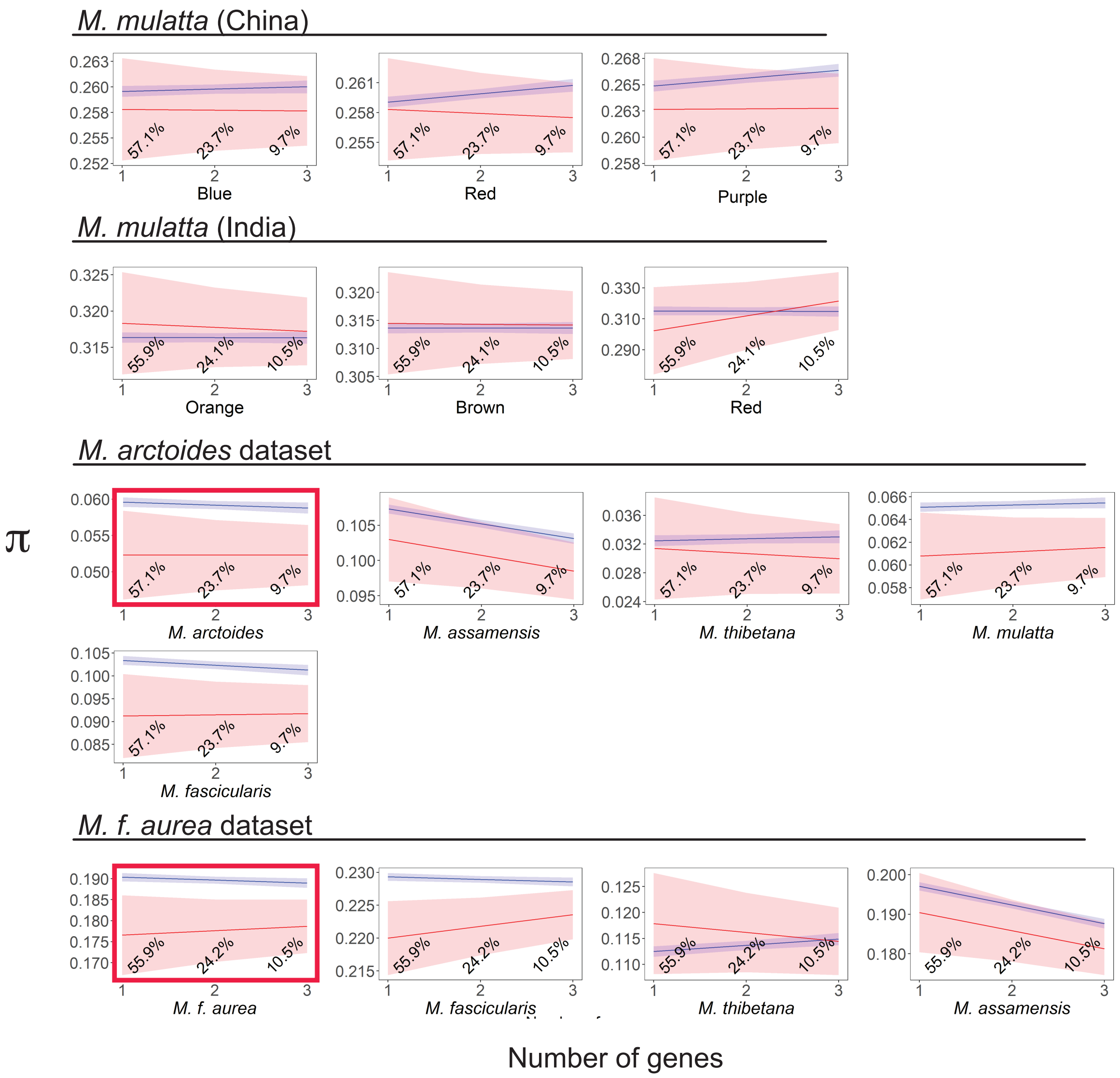

Fig. S6. In the *M. arctoides* and *M. f. aurea* datasets but in *M. mulatta* from China and India datasets the predicted values (marginal means) for most species or population of π in 100 kb windows are generally lower in $N_{interact}$ windows (red) compared non-$N_{interact}$ windows (blue) across biologically relevant numbers of genes in each window (Number of genes). Plotting, shading, and highlights follow Fig. 4.

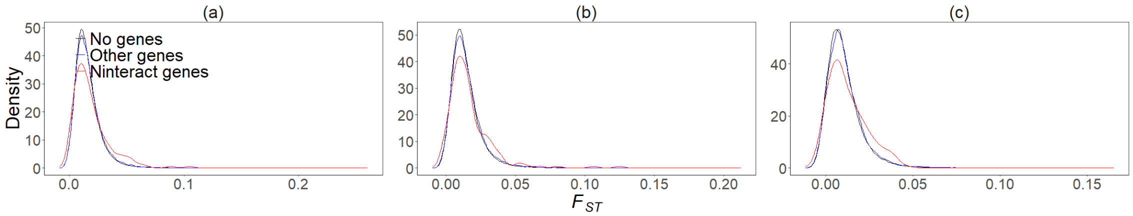

Fig. S7. Density plot of F_{ST} of wild *M. mulatta* from China for 30kb windows suggests some $N_{interact}$ windows (red) have atypically high F_{ST} compared to ROHs that contain only other genes (blue) or no genes (black). The order of panels matches Fig. S1.

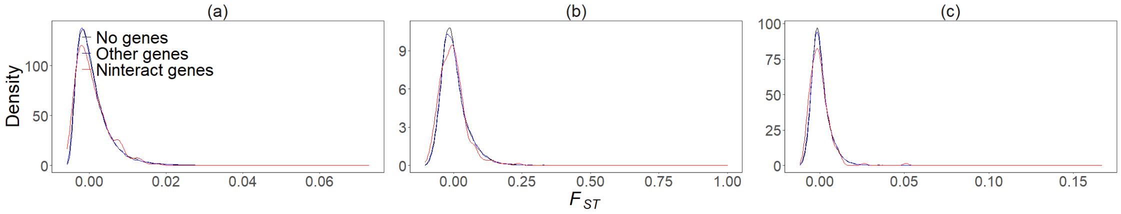

Fig. S8. Density plot of F_{ST} of captive *M. mulatta* from India for 30kb windows does not suggest that some $N_{interact}$ windows (red) have atypically high F_{ST} compared to ROHs that contain only other genes (blue) or no genes (black). The order of panels matches Fig. S2.

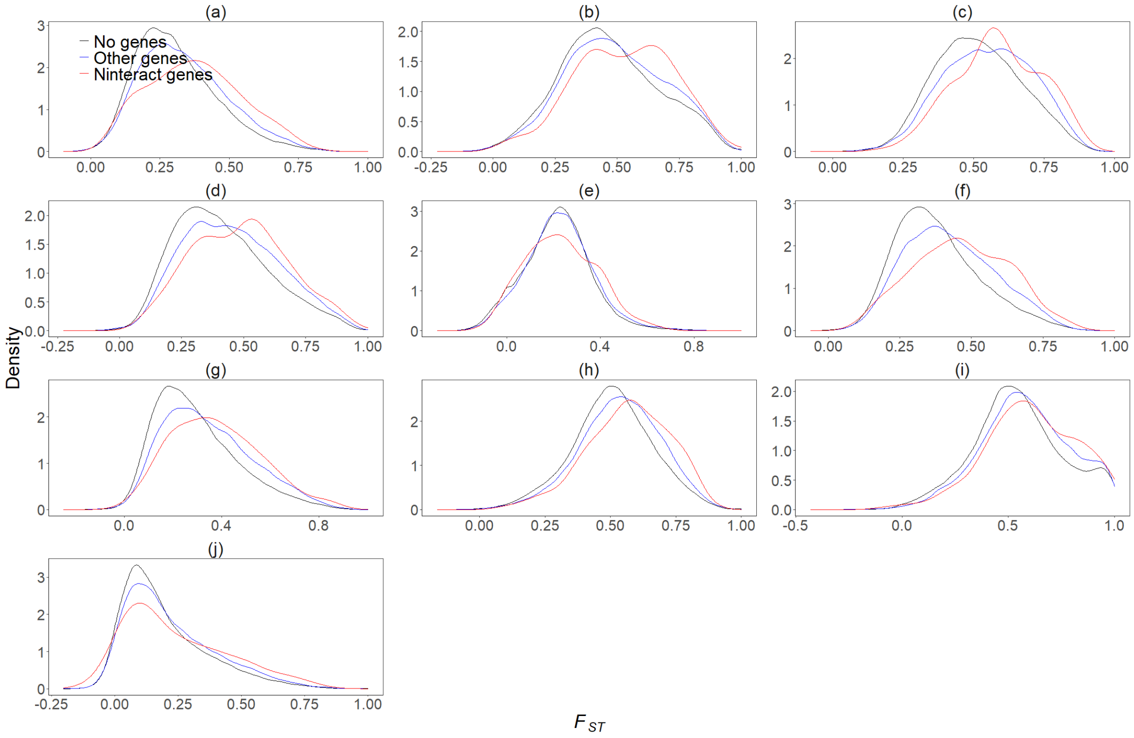

Fig. S9. Density plot of F_{ST} of *M. arctoides* dataset for 30kb windows suggests some $N_{interact}$ windows (red) have atypically high F_{ST} compared to ROHs that contain only other genes (blue) or no genes (black). The order of panels matches Fig. S3.

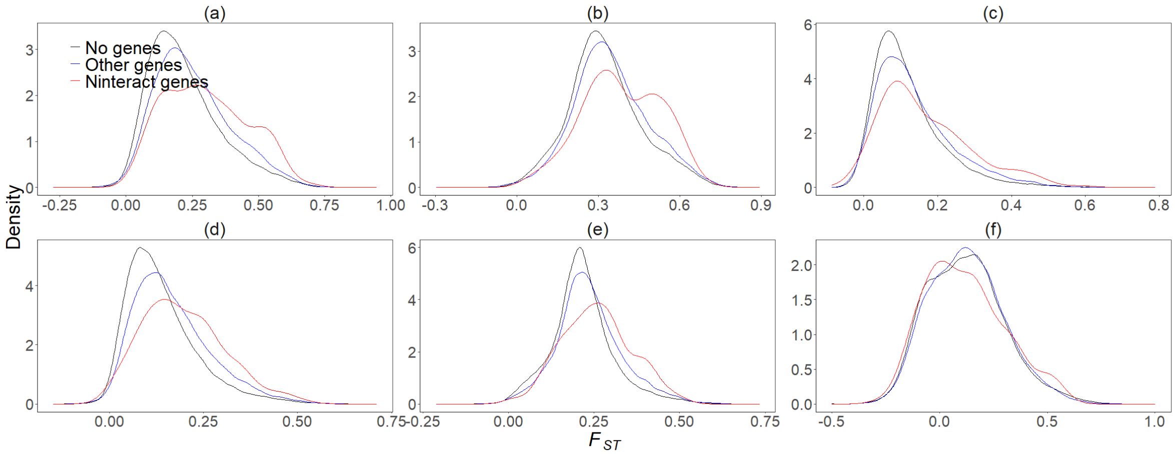

Fig. S10. Density plot of F_{ST} of *M. f. aurea* dataset for 30kb windows suggests some $N_{interact}$ windows (red) have atypically high F_{ST} compared to ROHs that contain only other genes (blue) or no genes (black). The order of panels matches Fig. S4.

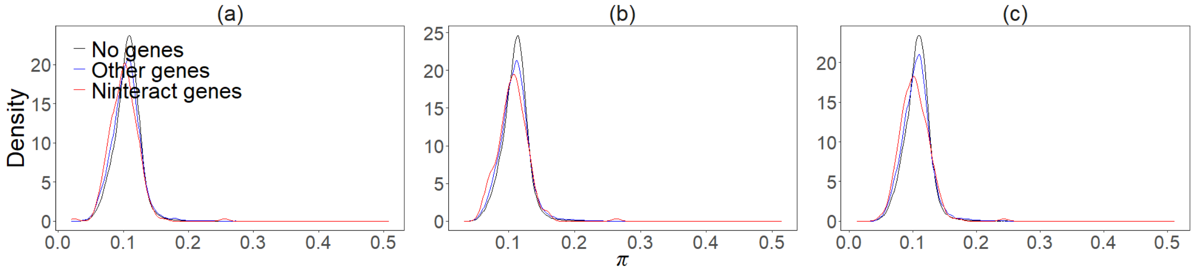

Fig. S11. Density plot of π for the wild *M. mulatta* from China for 30kb windows suggests some $N_{interact}$ windows (red) have atypically low π compared to ROHs that contain only other genes (blue) or no genes (black). The order of panels matches Fig. S1.

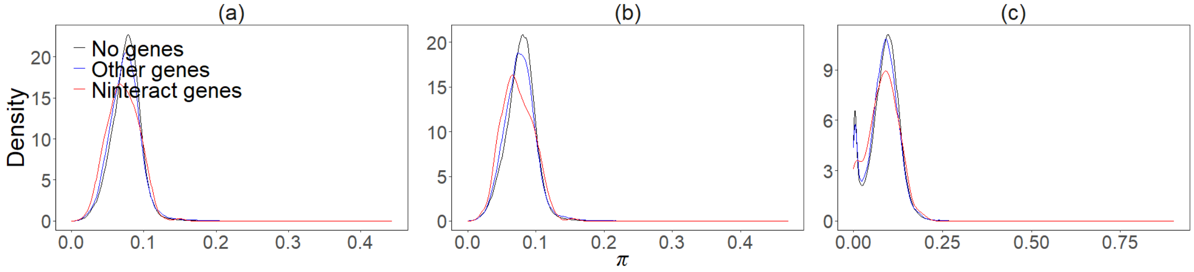

Fig. S12. Density plot of π for the captive *M. mulatta* from India for 30kb windows suggests some $N_{interact}$ windows (red) have atypically low π compared to ROHs that contain only other genes (blue) or no genes (black). The order of panels matches Fig. S2.

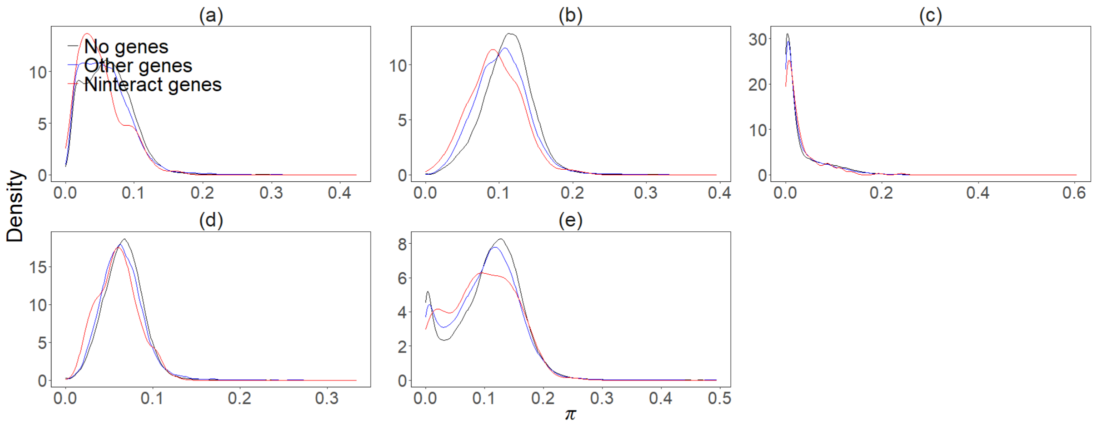

Fig. S13. Density plot of π for the *M. arctoides* dataset for 30kb windows suggests some $N_{interact}$ windows (red) have atypically low π compared to ROHs that contain only other genes (blue) or no genes (black). The order of panels matches Fig. S3.

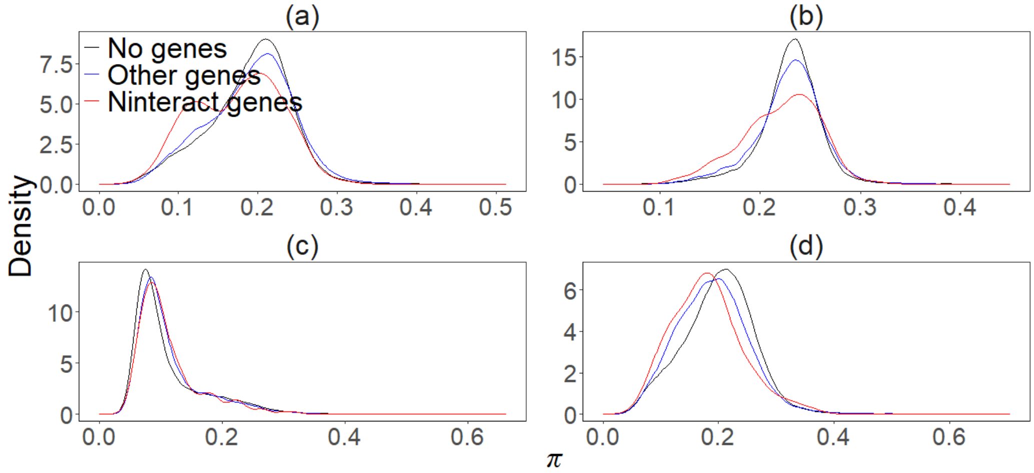

Fig. S14. Density plot of π for the *M. f. aurea* dataset for 30kb windows suggests some $N_{interact}$ windows (red) have atypically low π compared to ROHs that contain only other genes (blue) or no genes (black). The order of panels matches Fig. S4.

F_{ST} outlier analyses: wild macaques from China

Outlier analysis identified a significant excess of $N_{interact}$ windows that were upper F_{ST} outliers (comparisons between Blue and Purple: 14.1%, between Blue and Red: 10.4%, and between Purple and Red: 8.3%) compared to non-$N_{interact}$ windows (comparisons between Blue and Purple: 3.4%, between Blue and Red: 3.3%, and between Purple and Red: 3.3%; $P < 0.001$ for all three comparisons, binomial tests). The excess of upper outliers is illustrated in Fig. S15 by the red dots above the regression line.

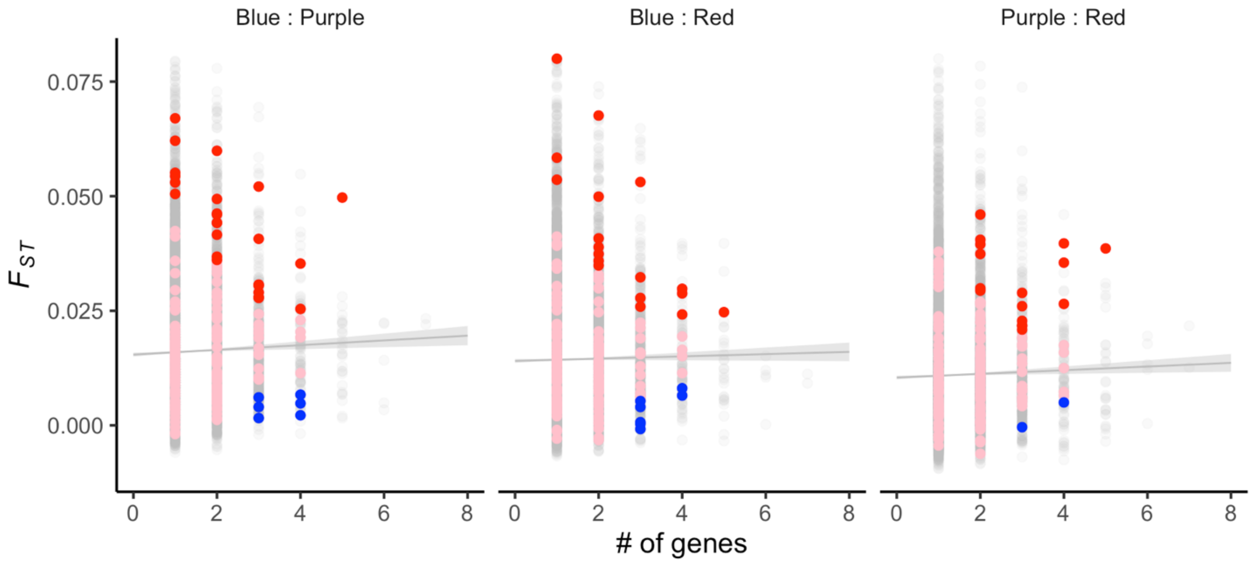

Fig. S15. F_{ST} outlier analysis for wild *M. mulatta* from China identifies a significant excess of upper F_{ST} outliers in $N_{interact}$ windows (red dots above fitted line) for all three pairwise comparisons. Blue dots indicate $N_{interact}$ windows with atypically low differentiation in each pairwise comparison. Pink dots are $N_{interact}$ windows that are not outliers; gray dots are non-$N_{interact}$ windows (including outliers and non-outliers).

F_{ST} outlier analysis: captive *M. mulatta* from India

Outlier analysis (Fig. S16) also identified an excess of $N_{interact}$ windows that were upper F_{ST} outliers (comparisons between Brown and Orange: 4.8%, between Brown and Red: 4.2%, and between Orange and Red: 4.8%) compared to non-$N_{interact}$ windows (comparisons between Brown and Orange: 3.5%, between Brown and Red: 3.3%, and between Orange and Red: 2.8%). However, this excess was not individually significant for these comparisons (*P* > 0.05; binomial tests).

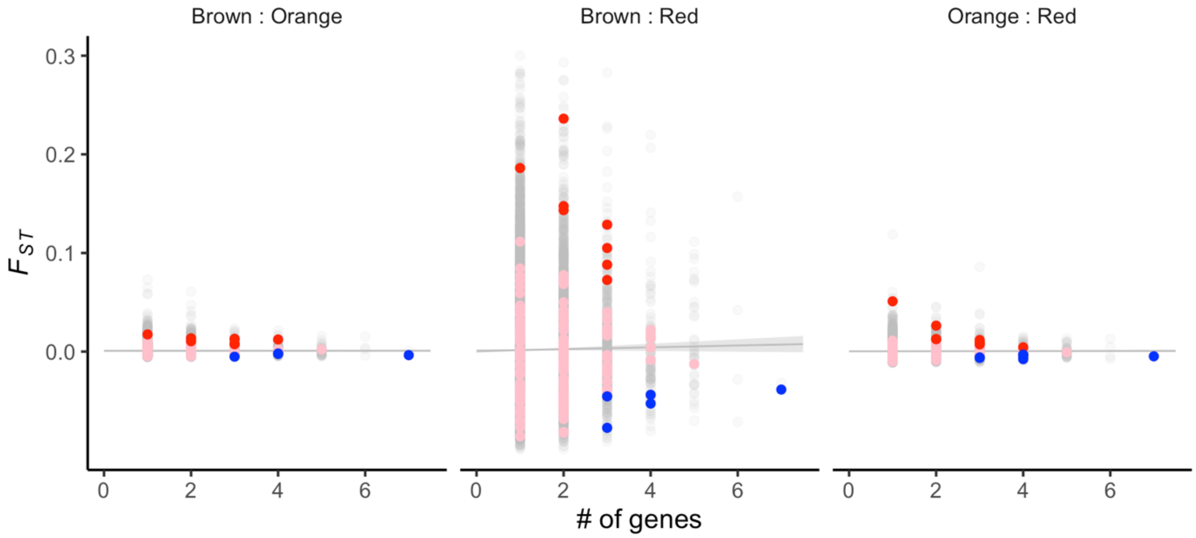

Fig. S16. F_{ST} outlier analysis of captive *M. mulatta* from India data identifies a significant excess of upper F_{ST} outliers in $N_{interact}$ windows (red dots above fitted line) in one of three comparisons (Orange : Red). Blue dots indicate $N_{interact}$ windows with atypically low differentiation in each pairwise comparison. Pink dots are $N_{interact}$ windows that are not outliers; gray dots are non-$N_{interact}$ windows (including outliers and non-outliers).

F_{ST} outlier analysis: *M. arctoides* dataset

For the *M. arctoides* dataset, ten pairwise comparisons were considered between five populations/species including two (*M. thibetana* and *M. fascicularis*) that had only one individual. Outlier analysis (Fig. S17) identified a significant excess of $N_{interact}$ windows that were upper F_{ST} outliers (comparisons between *M. arctoides* and *M. assamensis*: 7.7%, between *M. arctoides* and *M. fascicularis*: 7.7%, between *M. arctoides* and *M. mulatta*: 5.7%, between *M. arctoides* and *M. thibetana*: 4.6%, between *M. assamensis* and *M. fascicularis*: 6.7%, between *M. assamensis* and *M. mulatta*: 8.2%, between *M. assamensis* and *M. thibetana*: 9.3%, between *M. mulatta* and *M. fascicularis*: 10.8%, between *M. thibetana* and *M. fascicularis*: 7.7%, and between *M. thibetana* and *M. mulatta*: 7.2%) compared to non-$N_{interact}$ windows (2.5%, 2.1%, 1.2%, 1.5%, 2.5%, 2.1%, 2.4%, 3.4%, 1.4%, and 1.5%, respectively). This excess was individually significant for all comparisons ($P < 0.05$; binomial tests).

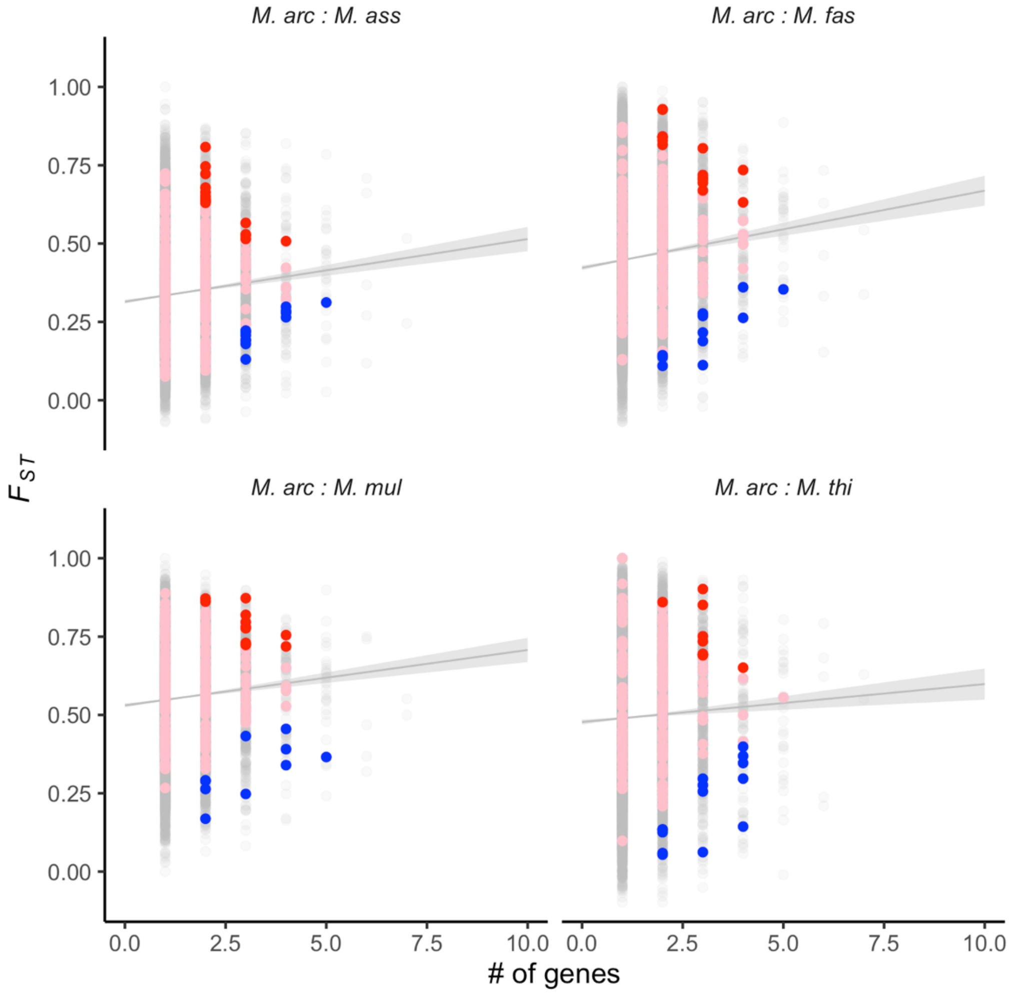

Fig. S17. F_{ST} outlier analysis for *M. arctoides* dataset identifies a significant excess of upper F_{ST} outliers in $N_{interact}$ windows (red dots above fitted line). Species names are abbreviated with first three letters; only pairwise comparisons involving the focal species *M. arctoides* are shown. Blue dots indicate $N_{interact}$ windows with atypically low differentiation in each pairwise comparison. Pink dots are $N_{interact}$ windows that are not outliers; gray dots are non- $N_{interact}$ windows (including outliers and non-outliers).

F_{ST} analysis; *M. f. aurea*

For the *M. f. aurea* dataset, three pairwise comparisons were considered between three groups including one (*M. thibetana* and *M. assamensis*) that is a group comprised of two closely related species. Outlier analysis (Fig. S18) identified a significant excess of $N_{interact}$ windows that were upper F_{ST} outliers (comparisons between *M. f. aurea* and *M. fascicularis*: 9.8%, between *M. f. aurea* and *M. thibetana* 5.2%, between *M. f. aurea* and *M. assamensis*: 7.2%, between *M. fascicularis* and *M. thibetana*: 6.2%, between *M. fascicularis* and *M. assamensis*: 9.2%, *and between M. thibetana* and *M. assamensis*: 7.7%) compared to non-$N_{interact}$ windows (3.5 %, 2.1 %, 2.5 %, 2.4 %, 3.0 %, and 2.3%, respectively). This excess was individually significant for all comparisons (P < 0.05; binomial tests).

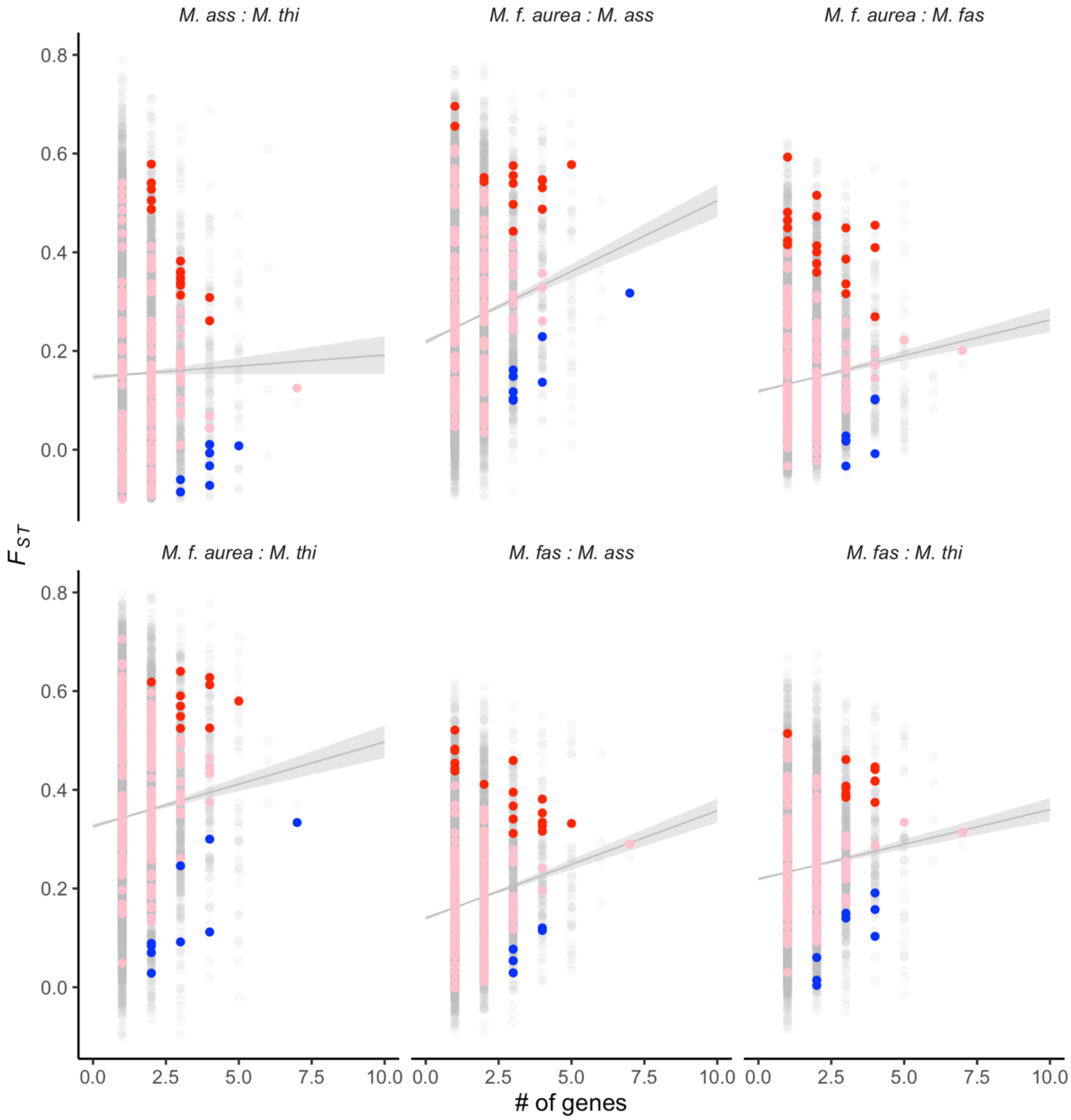

Fig. S18. F_{ST} outlier analysis for *M. f. aurea* data identifies a significant excess of upper F_{ST} outliers in $N_{interact}$ windows (red dots above fitted line). Blue dots indicate $N_{interact}$ windows with atypically low differentiation in each pairwise comparison. Pink dots are $N_{interact}$ windows that are not outliers; gray dots are non- $N_{interact}$ windows (including outliers and non-outliers).

π analysis; wild *M. mulatta* from China

For the three populations of wild *M. mulatta* from China defined based on mitochondrial clades, outlier analysis identified a significant excess of $N_{interact}$ windows that were lower π outliers (Blue: 4.6%, Red: 6.2%, and Purple: 5.7%) compared to non-$N_{interact}$ windows (Blue: 1.6%, Red: 1.5%, and Purple: 1.4%, binomial tests, $P < 0.01$, Fig. S19).

A

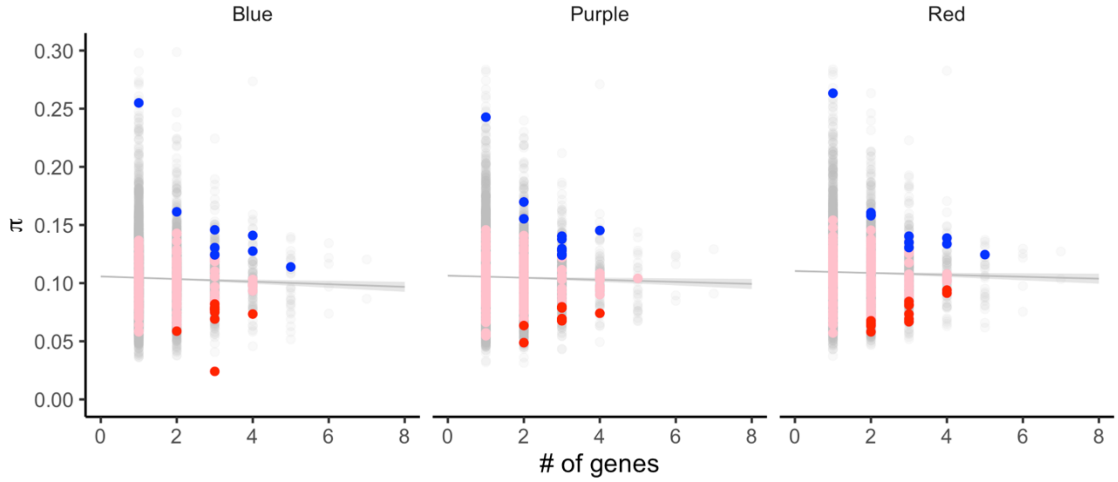

Fig. S19. π outlier analysis for wild *M. mulatta* from China identifies a significant excess of lower π outliers in $N_{interact}$ windows (red dots below fitted line). The very low value for π in the Blue group is a window that contains the $N_{interact}$ gene NDUFB8. Blue dots indicate $N_{interact}$ windows with atypically high diversity. Pink dots are $N_{interact}$ windows that are not outliers; gray dots are non- $N_{interact}$ windows (including outliers and non-outliers).

π analysis; captive *M. mulatta* from India

For the three populations of captive *M. mulatta* from India defined based on mitochondrial clades, outlier analysis identified a significant excess of $N_{interact}$ windows that were lower π outliers (Brown: 5.2%, Orange: 4.1%, and Red: 6.2%) compared to non-$N_{interact}$ windows (Brown: 1.5%, Orange: 1.6%, and Red: 2.1%, binomial tests, $P < 0.05$, Fig. S20).

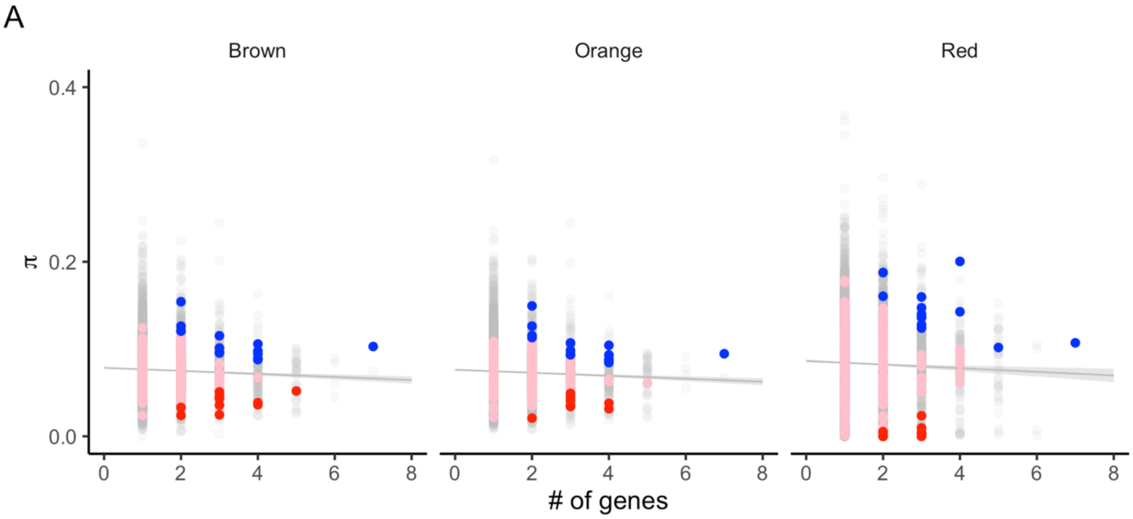

Fig. S20. π outlier analysis for captive *M. mulatta* from India identifies a significant excess of lower π outliers in $N_{interact}$ windows (red dots below fitted line). For the Red group, there are 11 lower π outliers, but many of them overlap in the plot. Blue dots indicate $N_{interact}$ windows with atypically high diversity. Pink dots are $N_{interact}$ windows that are not outliers; gray dots are non-$N_{interact}$ windows (including outliers and non-outliers).

π analysis; *M. arctoides* dataset

For *M. arctoides* dataset, outlier analysis identified a significant excess of $N_{interact}$ windows that were lower π outliers (*M arctoides*: 2.6%, *M. fascicularis*: 6.2%, *M. mulatta*: 8.2%, *M. thibetana*: 2.6%, *M. assamensis*: 8.2%) compared to non-$N_{interact}$ windows (0.1%, 1.6%, 1.2%, 0.2%, and 1.4%, respectively, binomial tests, $P < 0.05$, Fig. S21).

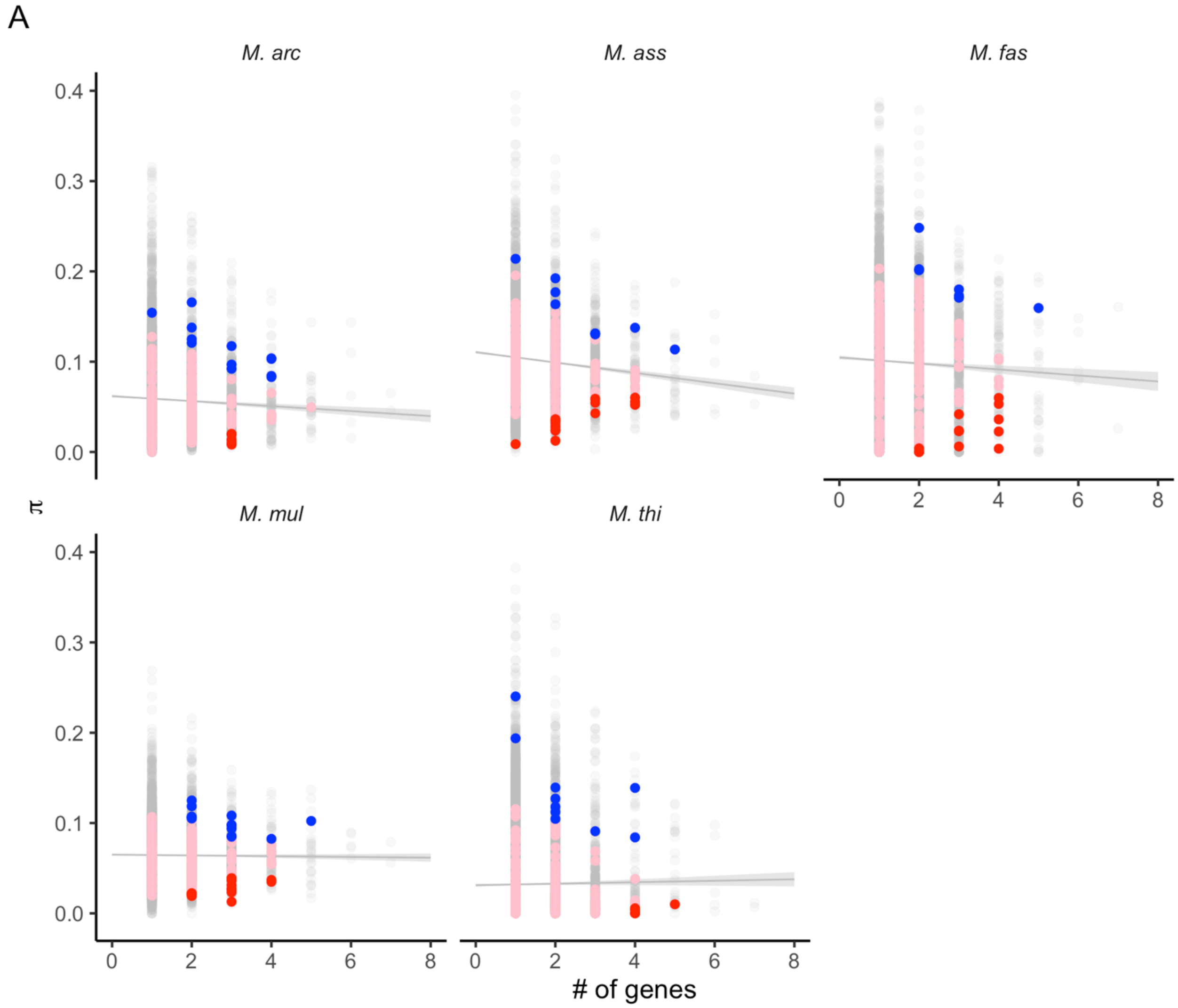

Fig. S21. π outlier analysis for the *M. arctoides* dataset identifies a significant excess of lower π outliers in $N_{interact}$ windows (red dots below fitted line). For *M. arctoides*, there are 5 lower π outliers, but many of them overlap in the plot. Blue dots indicate $N_{interact}$ windows with atypically high diversity. Pink dots are $N_{interact}$ windows that are not outliers; gray dots are non-$N_{interact}$ windows (including outliers and non-outliers).

π analysis; *M. f. aurea* dataset

For *M. f. aurea* dataset, outlier analysis identified a significant excess of $N_{interact}$ windows that were lower π outliers (*M. f. aurea*: 5.7%, *M. fascicularis*: 10.3%, *M. thibetana*: 4.1%, and *M. assamensis*: 4.6%) compared to non-$N_{interact}$ windows (1.9%, 2.7%, 0.7%, and 1.1%, respectively, binomial tests, P < 0.05, Fig. S22).

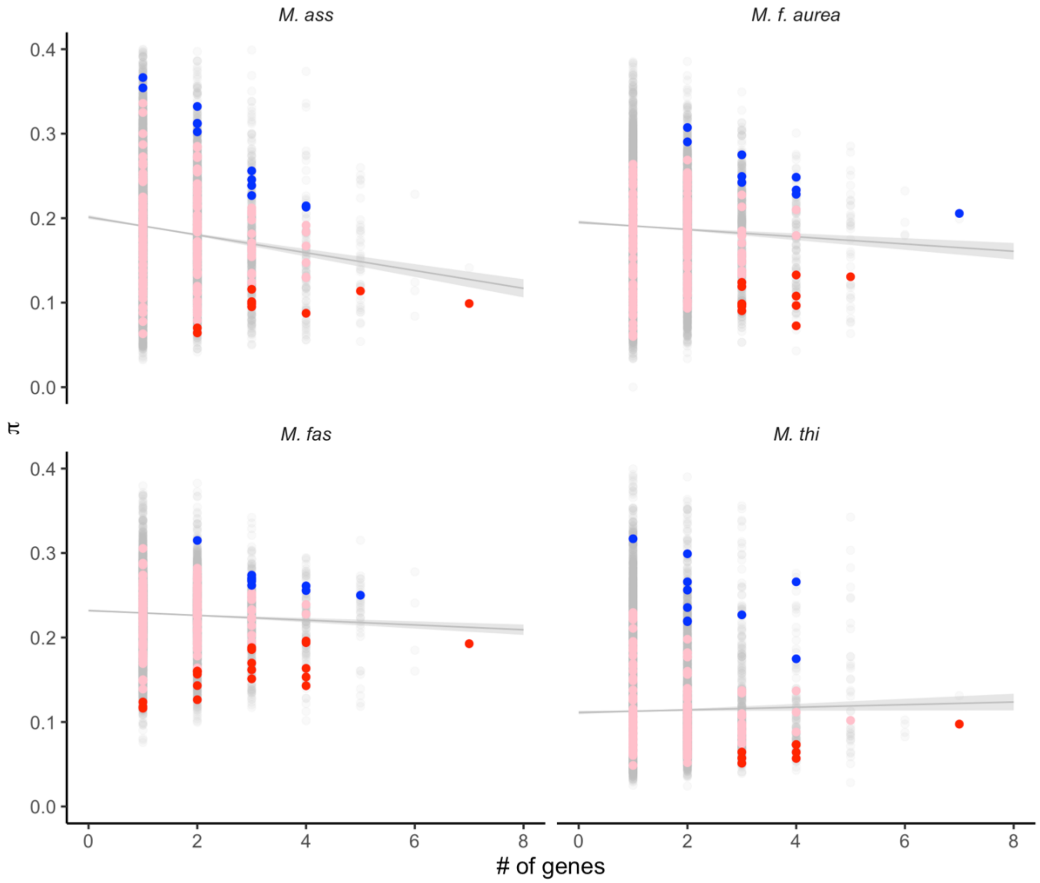

Fig. S22. π outlier analysis for *M. f. aurea* dataset identifies a significant excess of lower π outliers in $N_{interact}$ windows (red dots below fitted line). Blue dots indicate $N_{interact}$ windows with atypically high diversity. Pink dots are $N_{interact}$ windows that are not outliers; gray dots are non-$N_{interact}$ windows (including outliers and non-outliers).

F_{ST} outlier intersection: wild *M. mulatta* from China

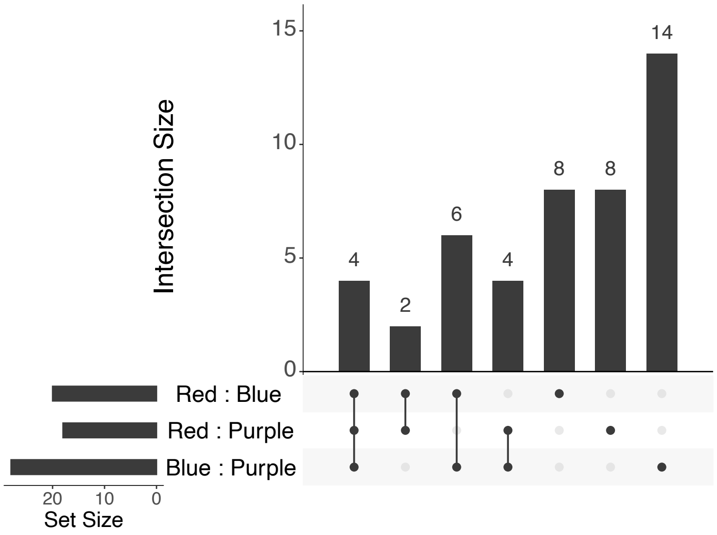

Fig. S23. Upset plot illustrating common outliers from the 30kb window F_{ST} analysis of the *M. mulatta* from China dataset. On the bottom left, the set size histogram illustrates the total number of outliers in each comparison; on the top, the intersection size illustrates the number of outliers that are shared across comparisons. For example, there are 19 F_{ST} outliers in the Red: Blue comparison (listed in Table S7); four of these are found in all three pairwise comparisons, seven are found only in this pairwise comparison.

F_{ST} outlier intersection: captive *M. mulatta* from India

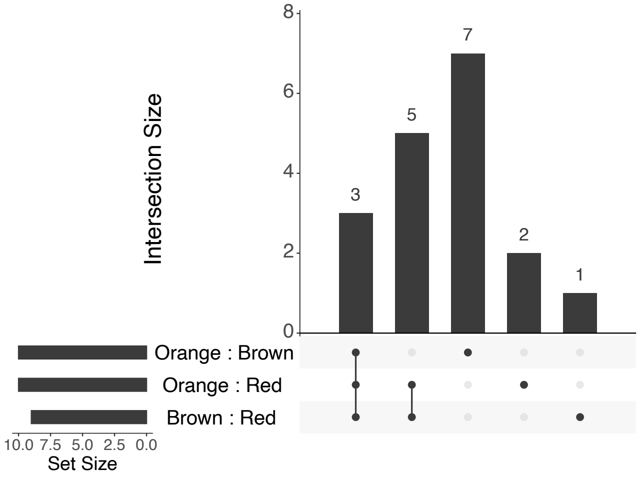

Fig. S24. Upset plot illustrating common outliers from the 30kb window F_{ST} analysis of the *M. mulatta* from India dataset. Labeling follows Fig. S23.

F_{ST} outlier intersection: *M. arctoides* dataset

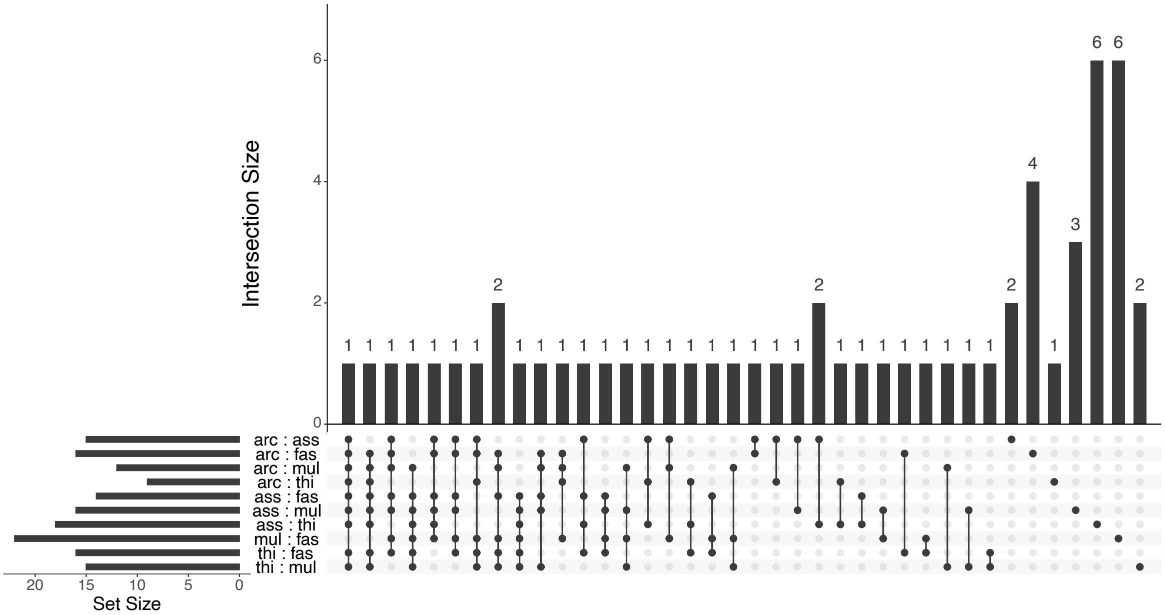

Fig. S25. Upset plot illustrating common outliers from the 30kb window F_{ST} analysis of the *M. arctoides* dataset. Labeling follows Fig. S23.

F_{ST} outlier intersection: *M. f. aurea* dataset

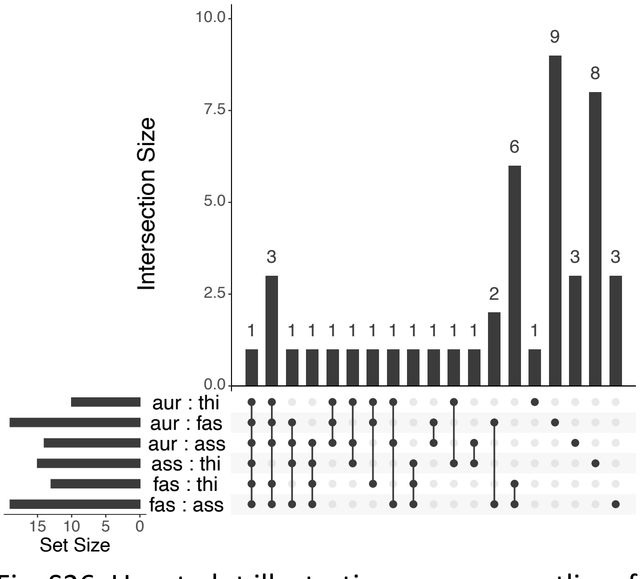

Fig. S26. Upset plot illustrating common outliers from the 30kb window F_{ST} analysis of the *M. f. aurea* dataset. Labeling follows Fig. S23.

π outlier intersection: wild *M. mulatta* from China

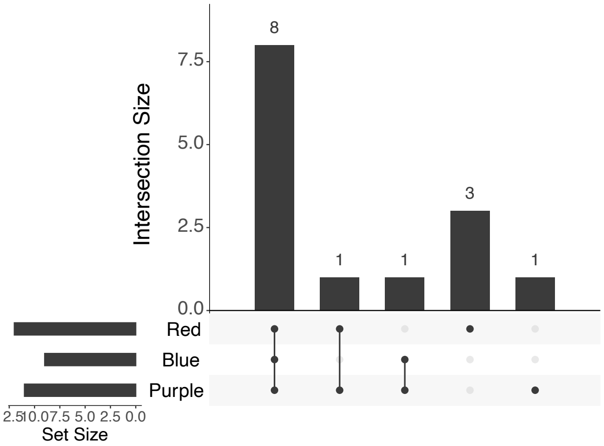

Fig. S27. Upset plot illustrating common outliers from the 30kb window π analysis of the *M. mulatta* from China dataset. Labeling follows Fig. S23.

π outlier intersection: captive *M. mulatta* from India

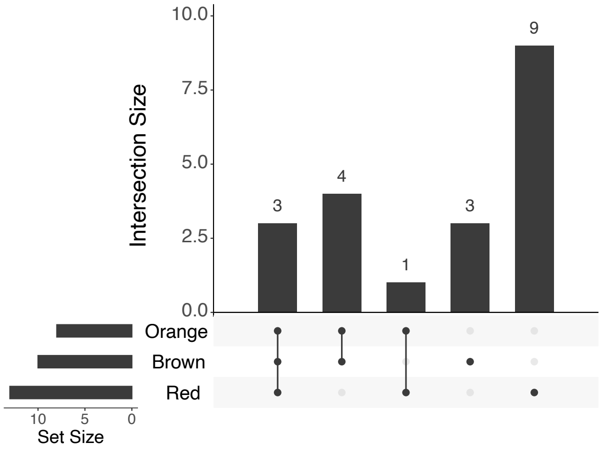

Fig. S28. Upset plot illustrating common outliers from the 30kb window π analysis of the *M. mulatta* from India dataset. Labeling follows Fig. S23.

π outlier intersection: *M. arctoides* dataset

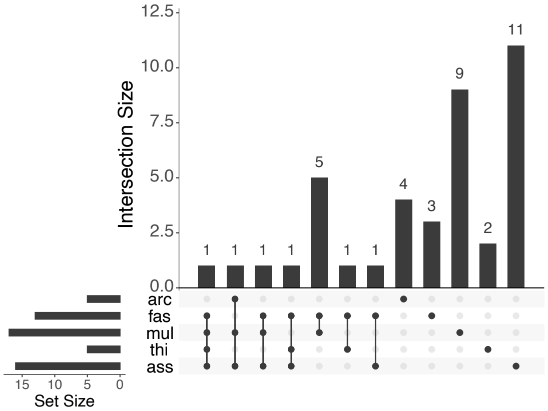

Fig. S29. Upset plot illustrating common outliers from the 30kb window π analysis of the *M. arctoides* dataset. Labeling follows Fig. S23.

π outlier intersection: *M. f. aurea* dataset

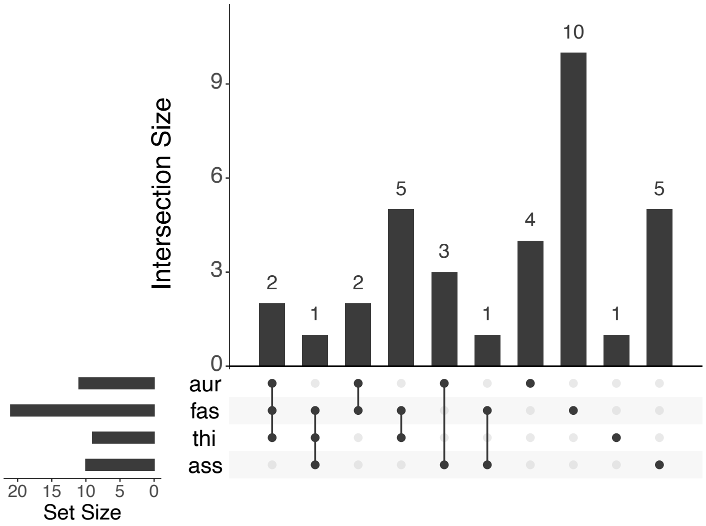

Fig. S30. Upset plot illustrating common outliers from the 30kb window π analysis of the *M. f. aurea* dataset. Labeling follows Fig. S23.

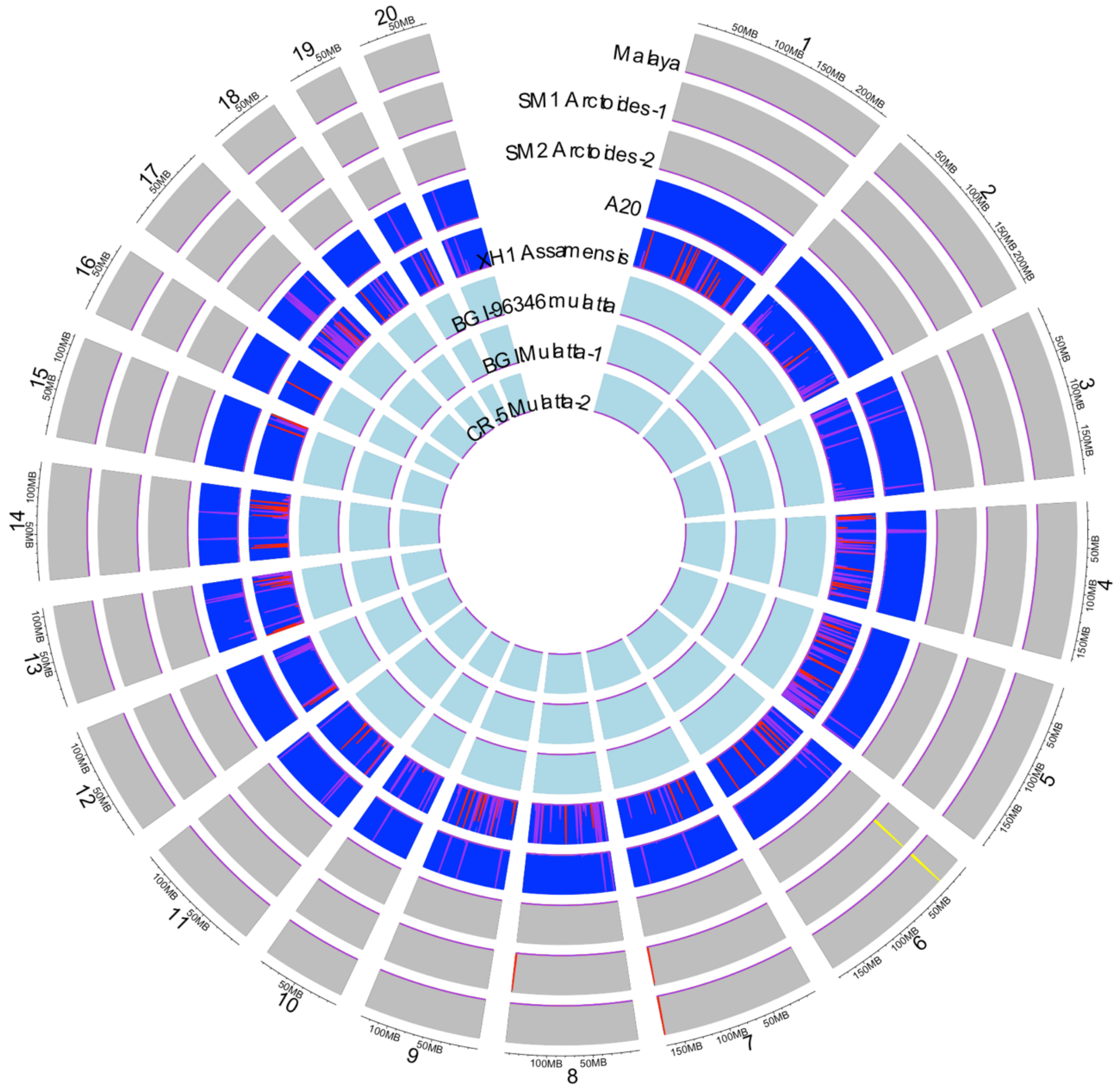

Fig S31. Admixfrog analysis of *M. arctoides*, *M. assamensis*, and *M. mulatta*. Gray, blue, light blue, red, yellow, and purple indicate homozygous *M. arctoides*, homozygous *M. assamensis*, homozygous *M. mulatta*, heterozygous *M. arctoides/M. assamensis*, heterozygous *M. arctoides/M. mulatta* and heterozygous *M. assamensis/M. mulatta* respectively. Numbers refer to chromosomes and individual genomes are in each layer. As detailed in the main text, most individuals do not have much evidence of recent gene flow with the exception of one *M. assamensis* individual where extensive gene flow with *M. arctoides* is evidenced by an abundance of heterozygous *M. arctoides/M. assamensis* blocks.

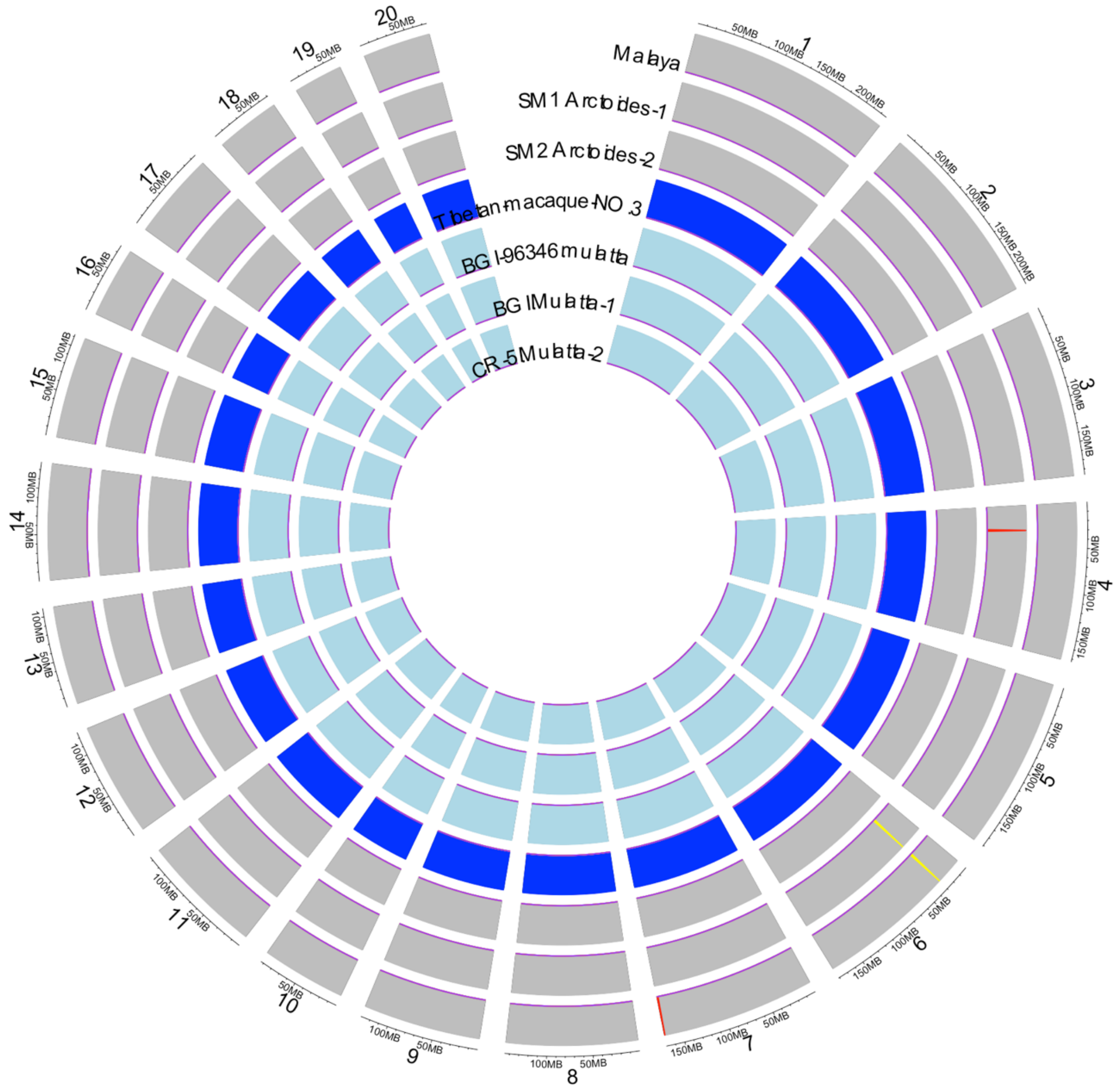

Fig S32. Admixfrog analysis of *M. arctoides*, *M. thibetana*, and *M. mulatta*. Gray, blue, light blue, red, yellow, and purple indicate homozygous *M. arctoides*, homozygous *M. assamensis*, homozygous *M. mulatta*, heterozygous *M. arctoides/M. thibetana*, heterozygous *M. arctoides/M. mulatta* and heterozygous *M. thibetana /M. mulatta* respectively. Numbers refer to chromosomes and individual genomes are in each layer.

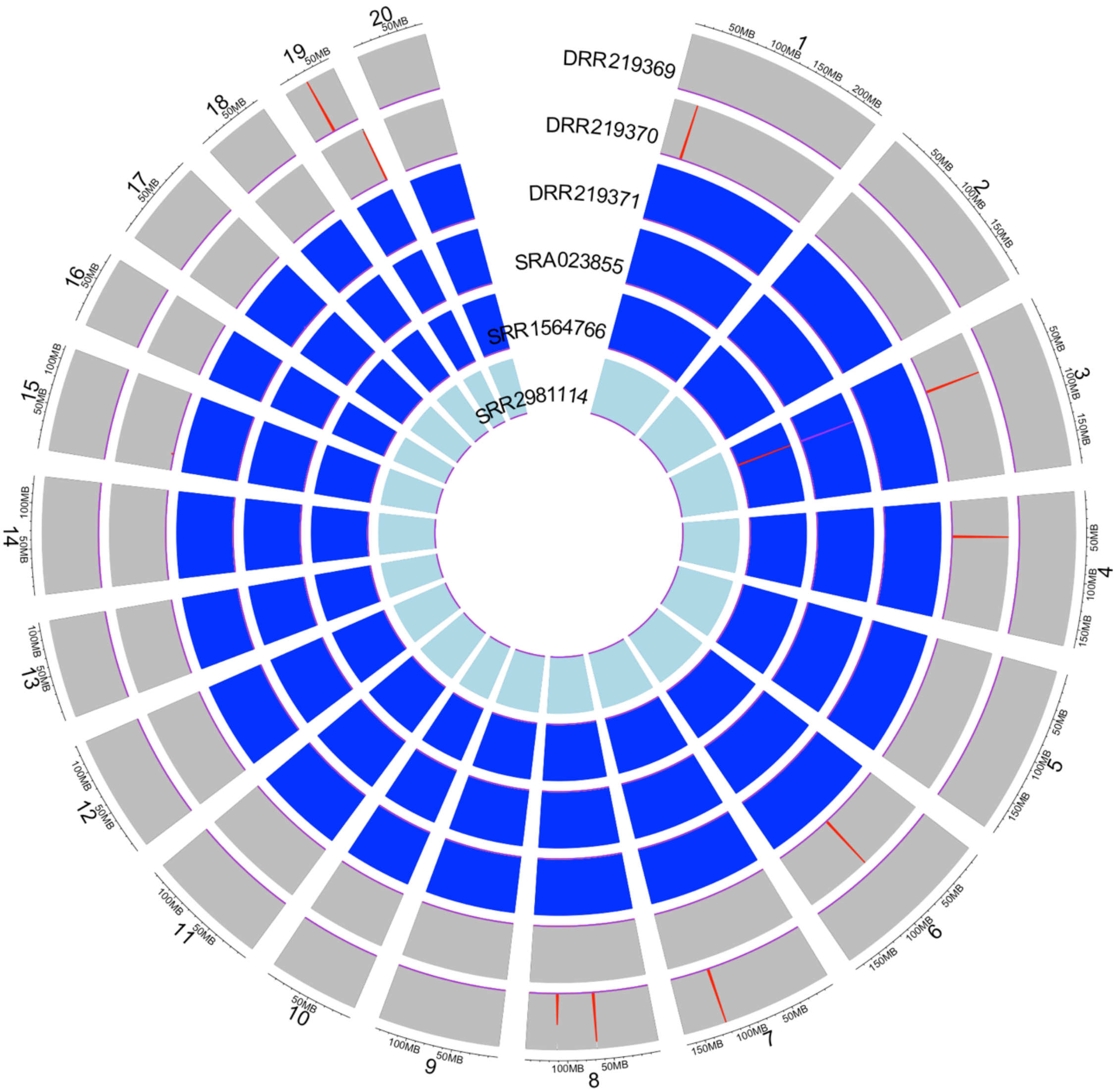

Fig S33. Admixfrog analysis of *M. f. aurea*, *M. fascicularis*, and *M. assamensis*. Gray, blue, light blue, red, yellow, and purple indicate homozygous *M. f. aurea*, homozygous *M. fascicularis*, homozygous *M. assamensis*, heterozygous *M. f. aurea /M. fascicularis*, heterozygous *M. f. aurea /M. assamensis* and heterozygous *M. fascicularis /M. assamensis* respectively. Numbers refer to chromosomes and individual genomes are in each layer.

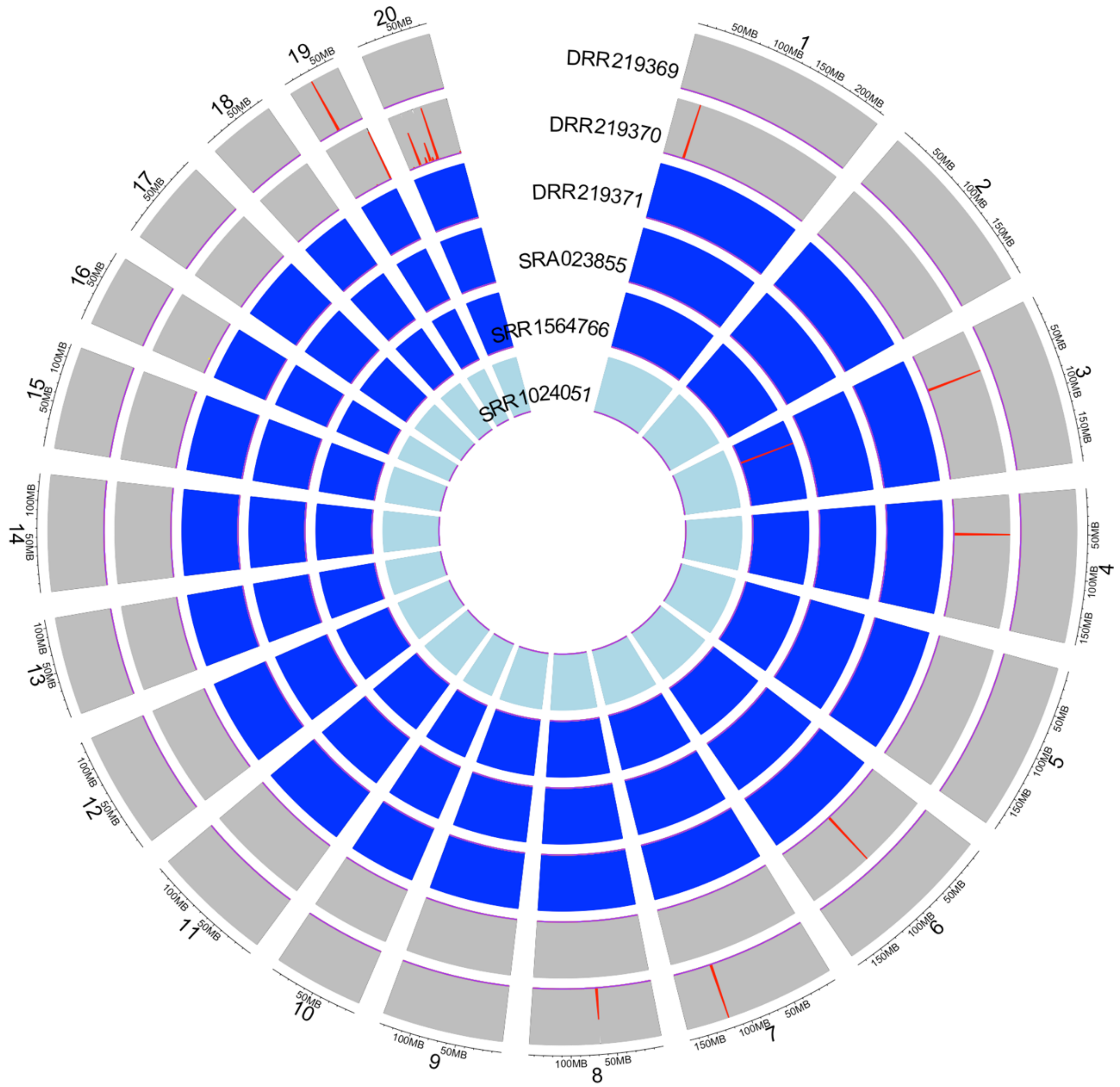

Fig S34. Admixfrog analysis of *M. f. aurea*, *M. fascicularis*, and *M. thibetana*. Gray, blue, light blue, red, yellow, and purple indicate homozygous *M. f. aurea*, homozygous *M. fascicularis*, homozygous *M. thibetana*, heterozygous *M. f. aurea /M. fascicularis*, heterozygous *M. f. aurea /M. thibetana* and heterozygous *M. fascicularis /M. thibetana* respectively. Numbers refer to chromosomes and individual genomes are in each layer.

Comparison to *silenus* group macaques

Signatures of natural selection were frequently concordant in independent comparisons involving different sets of species or populations in this study and also comparisons in the *silenus* and Sulawesi group species and populations (Evans et al. 2021). For example, NDUFA2, which is part of the N-module of OXPHOS complex I, was an upper F_{ST} outliers in at least four comparisons (F_{ST}) and a lower π outliers in at least four populations/species studied here

(Tables S7,S8,S10), and this gene was also an upper F_{ST} outlier in ten of ten pairwise comparisons in the *silenus* group and a lower π outliers in four of five species or populations in the *silenus* group (Evans et al. 2021). HARS2, which catalyzes the ATP-dependent ligation of histidine to the mitochondrial tRNA for this amino acid, was an outlier in many comparisons in this study (Tables S7,S8,S10) and also was an upper F_{ST} outlier in nine of ten pairwise comparisons in the *silenus* group and a lower π outlier in two of five species or populations in the *silenus* group (Evans et al. 2021). Similarly, MRPL55, which contributes to mitochondrial translation as a component of the large subunit of the mitochondrial ribosome, was an outlier in many comparisons in this study (Tables S7,S8,S10), and was an upper F_{ST} outlier in seven of ten pairwise comparisons in the *silenus* group and a lower π outlier in three of five species or populations examined (Evans et al. 2021). Although a lower π outlier in fewer than four or more species studied here, the REP gene POLRMT, which is involved with mitochondrial RNA polymerase and the initiation of mitochondrial replication, was an upper F_{ST} outlier in five comparisons in this study and also was an outlier for several metrics in *silenus* group macaques (Evans et al. 2021).

Examples of mitonuclear discordance in macaques

There are many examples of mitonuclear phylogenomic discordance in macaques. For example, mitochondrial DNA from *M. mulatta* is paraphyletic with respect to *M. fuscata* and *M. cyclopis*, that of *M. nemestrina* is paraphyletic with respect to *M. leonina*, *M. silenus* and the Sulawesi macaques, and mitochondrial relationships among Sulawesi macaques are poorly resolved whereas nuclear variation strongly supports reciprocal monophyly of species on the northern peninsula and species from the rest of Sulawesi (Fig. 1; Evans et al. 2017; Evans et al. 2020). Within *M. fascicularis*, Y chromosome variation has a sharp genetic discontinuity at the Isthmus of Kra whereas mitochondrial chromosome variation in the same individuals does not (Tosi et al. 2002). As detailed in the main text, there are also striking examples of introgression of diverged mitochondrial in *M. f. aurea* and *M. arctoides* (Tosi et al. 2003; Fan et al. 2018; Matsudaira et al. 2018) that also render evolutionary relationships among mitochondrial and nuclear genomes discordant.

References

Bouckaert, R., T. G. Vaughan, J. Barido-Sottani, S. Duchene, M. Fourment, A. Gavryushkina, J. Heled, G. Jones, D. Kuehnert, N. De Maio, M. Matschiner, F. K. Mendes, N. F. Mueller, H. A. Ogilvie, L. du Plessis, A. Popinga, A. Rambaut, D. Rasmussen, I. Siveroni, M. A. Suchard, C.-H. Wu, D. Xie, C. Zhang, T. Stadler, and A. J. Drummond. 2019. BEAST 2.5: An advanced software platform for Bayesian evolutionary analysis. PLoS Computational Biology 15:Article No.: e1006650.

Clutton-Brock, T. H. and D. Lukas. 2012. The evolution of social philopatry and dispersal in female mammals. Molecular Ecology 21:472–492.

Dierckxsens, N., P. Mardulyn, and G. Smits. 2017. NOVOPlasty: de novo assembly of organelle genomes from whole genome data. Nucleic Acids Research 45:e18.

Dittus, W. P. 1975. Population dynamics of the toque monkey, *Macaca sinica*. Socioecology and Psychology of Primates:125-151.

Dobson, F. S. 1982. Competition for mates and predominant juvenile male dispersal in mammals. Animal Behaviour 30:1183–1192.

Evans, B. J., M. T. Gansauge, M. W. Tocheri, M. A. Schillaci, T. Sutikna, Jatmiko, E. W. Saptomo, A. Klegarth, A. J. Tosi, D. J. Melnick, and M. Meyer. 2020. Mitogenomics of macaques (*Macaca*) across Wallace's Line in the context of modern human dispersals. J Hum Evol 146:102852.

Evans, B. J., B. M. Peter, D. J. Melnick, N. Andayani, J. Supriatna, J. Zhu, and A. J. Tosi. 2021. Mitonuclear interactions and introgression genomics of macaque monkeys (*Macaca*) highlight the influence of behaviour on genome evolution. Proceedings of the Royal Society B 288:20211756.

Evans, B. J., A. J. Tosi, K. Zeng, J. Dushoff, A. Corvelo, and D. J. Melnick. 2017. Speciation over the edge: Gene flow among non-human primate species across a formidable biogeographic barrier. Royal Society Open Science 4.

Fan, Z., A. Zhou, N. Osada, J. Yu, J. Jiang, P. Li, L. Du, L. Niu, J. Deng, H. Xu, and others. 2018. Ancient hybridization and admixture in macaques (genus *Macaca*) inferred from whole genome sequences. Molecular Phylogenetics and Evolution 127:376-386.

Fischer, J., J. P. Higham, S. C. Alberts, L. Barrett, J. C. Beehner, T. J. Bergman, A. J. Carter, A. Collins, S. Elton, J. Fagot, M. J. Ferreira Da Silva, K. Hammerschmidt, P. Henzi, C. J. Jolly, S. Knauf, G. H. Kopp, J. Rogers, C. Roos, C. Ross, R. M. Seyfarth, J. Silk, N. Snyder-Mackler, V. Staedele, L. Swedell, M. L. Wilson, and D. Zinner. 2019. Insights into the evolution of social systems and species from baboon studies. eLife 8:Article No.: e50989.

Greenwood, P. J. 1980. Mating systems, philopatry and dispersal in birds and mammals. Animal Behaviour 28:1140–1162.

Hamilton, W. D. and R. M. May. 1977. Dispersal in stable habitats. Nature 269:578-581.

Li, X.-Y. and H. Kokko. 2019. Sex-biased dispersal: a review of the theory. Biological Reviews (Cambridge) 94:721-736.

Matsudaira, K., Y. Hamada, S. Bunlungsup, T. Ishida, A. M. San, and S. Malaivijitnond. 2018. Whole mitochondrial genomic and Y-chromosomal phylogenies of Burmese long-tailed macaque (*Macaca fascicularis aurea*) suggest ancient hybridization between *fascicularis* and *sinica* species groups. Journal of Heredity 109:360–371.

Minh, B. Q., M. A. Nguyen, and A. von Haeseler. 2013. Ultrafast approximation for phylogenetic bootstrap. Molecular Biology and Evolution 30:1188-1195.

Mootha, V. K., J. Bunkenborg, J. V. Olsen, M. Hjerrild, J. R. Wisniewski, E. Stahl, M. S. Bolouri, H. N. Ray, S. Sihag, M. Kamal, N. Patterson, E. S. Lander, and M. Mann. 2003. Integrated analysis of protein composition, tissue diversity, and gene regulation in mouse mitochondria. Cell 115:629-640.

Nguyen, L. T., H. A. Schmidt, A. von Haeseler, and B. Q. Minh. 2015. IQ-TREE: A fast and effective stochastic algorithm for estimating maximum-likelihood phylogenies. Molecular Biology and Evolution 32:268–274.

Pusey, A. E. 1987. Dispersal and philopartry.250-266.

Roos, C., M. Kothe, D. M. Alba, E. Delson, and D. Zinner. 2019. The radiation of macaques out of Africa: Evidence from mitogenome divergence times and the fossil record. Journal of Human Evolution 133:114–132.

Sickmann, A., J. Reinders, Y. Wagner, C. Joppich, R. Zahedi, H. E. Meyer, B. Schoenfisch, I. Perschil, A. Chacinska, B. Guiard, P. Rehling, N. Pfanner, and C. Meisinger. 2003. The proteome of Saccharomyces cerevisiae mitochondria. Proceedings of the National Academy of Sciences of the United States of America 100:13207-13212.

Smits, P., J. Smeitink, and L. van den Heuvel. 2010. Mitochondrial Translation and Beyond: Processes Implicated in Combined Oxidative Phosphorylation Deficiencies. Journal of Biomedicine & Biotechnology:Article No.: 737385.

Swedell, L. 2010. African papionins: Diversity of social organization and ecological flexibility. Primates in Perspective.

Thierry, B. 2007. The macaques: a double-layered social organisation. Pp. 224-239 *in* C. J. Campbell, A. Fuentes, K. C. MacKinnon, N. Panger, and S. K. Bearder, eds. Primates in Perspective. Oxford University Press, New York.

Tosi, A. J., J. C. Morales, and D. J. Melnick. 2002. Y-chromosome and mitochondrial markers in Macaca fascicularis indicate introgression with Indochinese M. mulatta and a biogeographic barrier in the Isthmus of Kra. International Journal of Primatology 23:161–178.

Tosi, A. J., J. C. Morales, and D. J. Melnick. 2003. Paternal, maternal, and biparental molecular markers provide unique windows onto the evolutionary history of macaque monkeys. Evolution 57:1419–1435.

Warren, W. C., R. A. Harris, M. Haukness, I. T. Fiddes, S. C. Murali, J. Fernandes, P. C. Dishuck, J. M. Storer, M. Raveendran, and L. W. Hillier. 2020. Sequence diversity analyses of an improved rhesus macaque genome enhance its biomedical utility. Science 370.

Table S1. Results of linear models and permutation tests for runs of homozygosity. Coefficients and 95% confidence intervals in parentheses for the parameters of the linear model (see Methods for definitions of parameters) are mostly significant (*) but permutation test P values are generally not individually significant. $N_{interact}$ ROHs are significantly longer than non-$N_{interact}$ ROHs across all linear models and across all permutation tests (see main text).

Population	$N_{interact}$	*Number_of_genes*	*Interaction*	Permutation P value
M. mulatta from China				
Blue	0.730 (±0.034)*	0.228 (±0.002)*	-0.147 (±0.003)*	0.793
Red	0.512 (±0.047)*	0.283 (±0.004)*	-0.170 (±0.008)*	0.310
Purple	0.696 (±0.041)*	0.243 (±0.002)*	-0.149 (±0.004)*	0.966
M. mulatta from India				
Orange	0.834 (±0.025)*	0.244 (±0.001)*	-0.174(±0.002)*	0.784
Brown	0.722 (±0.079)*	0.261 (±0.005)*	-0.167 (±0.009)*	0.102
Red	0.274 (±0.262)*	0.351 (±0.018)*	-0.081 (±0.062)*	0.236
M. arctoides dataset				
M. arctoides	0.637 (±0.131)*	0.183 (±0.005)*	-0.097 (±0.012)*	0.055
M. assamensis	0.603 (±0.140)*	0.256 (±0.009)*	-0.146 (±0.019)*	0.237
M. thibetana	0.594 (±0.224)*	0.097 (±0.006)*	-0.040 (±0.013)*	0.307
M. mulatta	1.113 (±0.118)*	0.198 (±0.005)*	-0.155 (±0.007)*	0.000*

M. fascicularis	0.509 (±0.264)*	0.279 (±0.016)*	-0.120 (±0.044)*	0.746
M. f. aurea dataset				
M. f. aureus	0.426 (±0.234)*	0.266 (±0.010)*	-0.097 (±0.030)*	0.873
M. fascicularis	0.563 (±0.182)*	0.339 (±0.011)*	-0.185 (±0.025)*	0.771
M. thibetana	0.543 (±0.237)*	0.197 (±0.010)*	-0.097 (±0.027)*	0.734
M. assamensis	0.371 (±0.296)*	0.393 (±0.024)*	-0.128 (±0.082)*	0.057

*Individually significant (P < 0.05)

Table S2. Results of linear models and permutation tests for F_{ST} analysis in 30kb genomic windows. For the linear model, coefficients are followed by block bootstrap 95% confidence intervals in parentheses (see Methods for definitions of parameters). Significant values are indicated by asterisks; for some values small but non-zero values are reported as zero due to rounding to three decimal places.

	Comparison	$N_{interact}$	*gene_number*	*Interaction*	Permutation P value
M. mulatta from China					
	Blue : Purple	-0.001 (±0.005)	0.000(±0.000)*	0.002 (±0.003)	0.000*
	Blue : Red	0.001 (±0.005)	0.000(±0.000)	0.000 (±0.003)	0.007*
	Purple : Red	-0.002 (±0.004)	0.000(±0.000)*	0.002 (±0.002)	0.037*
M. mulatta from India					
	Brown : Orange	-0.000 (±0.001)	0.000(±0.000)	0.000 (±0.001)	0.661
	Brown : Red	-0.007 (±0.013)	0.001(±0.002)	0.000 (±0.008)	0.907
	Orange : Red	0.000 (±0.002)	0.000(±0.000)	-0.000 (±0.001)	0.925
M. arctoides dataset					
	M. arctoides : M. assamensis	0.084 (±0.054)*	0.020(±0.005)*	-0.032 (±0.020)*	0.010*
	M. arctoides : M. thibetana	0.082 (±0.064)*	0.012(±0.006)*	-0.028 (±0.032)	0.015*
	M. arctoides : M. mulatta	0.047 (±0.050)	0.018(±0.005)*	-0.013 (±0.027)	0.004*
	M. arctoides : M. fascicularis	0.049 (±0.064)	0.025(±0.006)*	-0.016 (±0.029)	0.041*
	M. assamensis : M. thibetana	-0.026 (±0.048)	0.002(±0.005)	0.017 (±0.028)	0.310
	M. assamensis : M. mulatta	0.034 (±0.056)	0.021(±0.006)*	-0.004 (±0.028)	0.000*
	M. assamensis : M. fascicularis	0.025 (±0.053)	0.030(±0.006)*	-0.005 (±0.029)	0.037*
	M. thibetana : M. mulatta	0.022 (±0.047)	0.008(±0.005)*	0.003 (±0.029)	0.004*
	M. thibetana : M. fascicularis	0.010 (±0.063)	0.014(±0.006)*	0.005 (±0.037)	0.070
	M. mulatta : M. fascicularis	0.020 (±0.063)	0.019(±0.005)*	-0.004 (±0.029)	0.111
M. f. aurea dataset					
	M. f. aureus : M. assamensis	0.060 (±0.049)*	0.028(±0.006)*	-0.015 (±0.025)	0.000*

M. f. aureus : M. thibetana	0.040 (±0.046)	0.017(±0.005)*	-0.006 (±0.028)	0.001*
M. f. aureus : M. fascicularis	0.047 (±0.034)*	0.014(±0.004)*	-0.014 (±0.020)	0.003*
M. fascicularis : M. assamensis	0.035 (±0.035)*	0.022(±0.004)*	-0.010 (±0.016)	0.001*
M. fascicularis : M. thibetana	0.019 (±0.032)	0.014(±0.004)*	-0.003 (±0.016)	0.012*
M. assamensis : M. thibetana	-0.030 (±0.058)	0.004(±0.005)*	0.011 (±0.032)	0.752

*Individually significant (P < 0.05)

Table S3. Results of linear models and permutation tests for F_{ST} analysis in 100kb genomic windows. For the linear model, coefficients are followed by block bootstrap 95% confidence intervals in parentheses (see Methods for definitions of parameters). Significant values are indicated by asterisks; for some values small but non-zero values are reported as zero due to rounding to three decimal places.

	Comparison	$N_{interact}$	*gene_number*	*Interaction*	Permutation P value
M. mulatta from China					
	Blue : Purple	0.002 (±0.003)	0.000(±0.000)*	-0.000 (±0.000)	0.006*
	Blue : Red	0.002 (±0.002)	0.000(±0.000)*	-0.000 (±0.000)	0.011*
	Purple : Red	0.002 (±0.002)	0.000(±0.000)*	-0.000 (±0.001)	0.009*
M. mulatta from India					
	Brown : Orange	-0.000 (±0.001)	0.000(±0.000)	-0.000 (±0.000)	0.978
	Brown : Red	-0.001 (±0.009)	0.001(±0.001)	-0.001 (±0.004)	0.900
	Orange : Red	0.000 (±0.001)	0.000(±0.000)	-0.000 (±0.000)	0.938
M. arctoides dataset					
	M. arctoides : M. assamensis	0.037 (±0.040)	0.008(±0.002)*	-0.002 (±0.011)	0.000*
	M. arctoides : M. thibetana	0.042 (±0.039)	0.005(±0.003)*	-0.001 (±0.012)	0.000*
	M. arctoides : M. mulatta	0.047 (±0.034)*	0.006(±0.003)*	-0.005 (±0.009)	0.000*
	M. arctoides : M. fascicularis	0.063 (±0.046)*	0.010(±0.003)*	-0.008 (±0.010)	0.000*
	M. assamensis : M. thibetana	0.005 (±0.031)	0.003(±0.002)*	0.001 (±0.008)	0.114
	M. assamensis : M. mulatta	0.041 (±0.037)*	0.009(±0.002)*	-0.004 (±0.010)	0.000*
	M. assamensis : M. fascicularis	0.049 (±0.035)*	0.013(±0.003)*	-0.006 (±0.012)	0.002*
	M. thibetana : M. mulatta	0.031 (±0.031)	0.004(±0.002)*	-0.001 (±0.011)	0.001*
	M. thibetana : M. fascicularis	0.044 (±0.042)	0.007(±0.003)*	-0.003 (±0.013)	0.001*
	M. mulatta : M. fascicularis	0.044 (±0.047)	0.008(±0.002)*	-0.004 (±0.011)	0.002*
M. f. aurea dataset					
	M. f. aureus : M. assamensis	0.047 (±0.038)*	0.012(±0.002)*	-0.006 (±0.009)	0.000*
	M. f. aureus : M. thibetana	0.026 (±0.036)	0.007(±0.002)*	-0.001 (±0.009)	0.003*
	M. f. aureus : M. fascicularis	0.042 (±0.027)*	0.006(±0.002)*	-0.007 (±0.008)	0.008*
	M. fascicularis : M. assamensis	0.032 (±0.028)*	0.009(±0.002)*	-0.005 (±0.006)	0.003*
	M. fascicularis : M. thibetana	0.017 (±0.023)	0.006(±0.002)*	-0.001 (±0.006)	0.005*
	M. assamensis : M. thibetana	-0.019 (±0.045)	0.003(±0.002)*	0.007 (±0.008)	0.355

*Individually significant ($P < 0.05$)

Table S4. Results of linear models and permutation tests for π analysis in 30kb genomic windows. For the linear model, coefficients are followed by block bootstrap 95% confidence intervals in parentheses (see Methods for definitions of parameters). Significant values are indicated by asterisks; for some values small but non-zero values are reported as zero due to rounding to three decimal places.

Population	$N_{interact}$	*gene_number*	*Interaction*	Permutation P value
M. mulatta from China				
Blue	-0.003 (±0.008)	-0.001 (±0.001)*	0.000 (±0.003)	0.042*
Red	-0.003 (±0.008)	-0.001 (±0.001)*	0.000 (±0.003)	0.058
Purple	-0.001 (±0.009)	-0.001 (±0.001)*	-0.001 (±0.004)	0.074
M. mulatta from India				
Orange	-0.005 (0.003)	-0.002 (0.000)*	0.002 (0.002)	0.120
Brown	-0.006 (0.004)	-0.002 (0.000)*	0.002 (0.002)	0.093
Red	-0.007 (0.007)	-0.002 (0.001)*	0.003 (0.004)	0.354
M. arctoides dataset				
M. arctoides	-0.011 (±0.012)	-0.003 (±0.001)*	0.003 (±0.005)	0.332
M. assamensis	-0.004 (±0.010)	-0.006 (±0.001)*	-0.001 (±0.005)	0.071
M. thibetana	0.003 (±0.014)	0.001 (±0.001)	-0.004 (±0.006)	0.049*
M. mulatta	-0.006 (±0.008)	-0.000 (±0.001)	0.001 (±0.004)	0.062
M. fascicularis	-0.005 (±0.018)	-0.003 (±0.002)*	0.001 (±0.008)	0.013*
M. f. aurea dataset				
M. f. aureus	-0.022 (±0.020)*	-0.004 (±0.002)*	0.005 (±0.007)	0.001*
M. fascicularis	-0.012 (±0.012)	-0.003 (±0.001)*	0.003 (±0.005)	0.001*
M. thibetana	0.008 (±0.014)	0.002 (±0.002)	-0.006 (±0.008)	0.254
M. assamensis	-0.006 (±0.016)	-0.011 (±0.002)*	0.002 (±0.009)	0.071

*Individually significant (P < 0.05)

Table S5. Results of linear models and permutation tests for π analysis in 100kb genomic windows. For linear models, coefficients are followed by 95% confidence intervals in parentheses (see Methods for definitions of parameters). π is significantly higher in $N_{interact}$ windows compared to non-$N_{interact}$ windows across all linear models and across all permutation tests (See main text). Significant values are indicated by asterisks; for some values small but non-zero values are reported as zero due to rounding to three decimal places.

Population	$N_{interact}$	*gene_number*	*Interaction*	Permutation P value
M. mulatta from China				
Blue	-0.002 (±0.006)	0.000 (±0.000)	-0.000 (±0.001)	0.096
Red	-0.002 (±0.004)	0.000 (±0.000)*	-0.000 (±0.001)	0.154
Purple	-0.003 (±0.005)	0.000 (±0.000)*	-0.000 (±0.001)	0.429
M. mulatta from India				
Orange	-0.003 (±0.005)	-0.000 (±0.000)	0.001 (±0.001)	0.036*
Brown	-0.003 (±0.005)	-0.000 (±0.000)	0.001 (±0.001)	0.010*
Red	-0.008 (±0.009)	-0.001 (±0.001)*	0.003 (±0.002)*	0.015*
M. arctoides dataset				
M. arctoides	-0.008 (±0.006)*	-0.000 (±0.000)	0.000 (±0.002)	0.001*
M. assamensis	-0.004 (±0.008)	-0.002 (±0.001)*	-0.000 (±0.002)	0.003*
M. thibetana	-0.000 (±0.010)	0.000 (±0.001)	-0.001 (±0.002)	0.074
M. mulatta	-0.004 (±0.006)	0.000 (±0.000)	0.000 (±0.001)	0.004*
M. fascicularis	-0.013 (±0.012)*	-0.001 (±0.001)*	0.001 (±0.003)	0.003*
M. f. aurea dataset				
M. f. aureus	-0.016 (±0.015)	-0.001 (±0.001)	0.002 (±0.003)	0.001*
M. fascicularis	-0.011 (±0.008)*	-0.000 (±0.001)	0.002 (±0.002)	0.016*
M. thibetana	0.008 (±0.010)	0.001 (±0.001)*	-0.003 (±0.003)*	0.342
M. assamensis	-0.007 (±0.012)	-0.005(±0.001)*	0.000(±0.004)	0.020*

*Individually significant (P < 0.05)

Table S6. Results of analysis of Tajima's *D* in 30kb and 100 kb genomic windows for each *M. mulatta* dataset. Population names match mitochondrial clades as indicated in the main text. Asterisks indicate individually significant P values based on permutation tests.

30 kb windows	Population	$N_{interact}$	non-$N_{interact}$	Difference	Permutation P value
	Chinese				
	Blue	-0.974	-0.910	-0.064	0.022*
	Purple	-0.790	-0.744	-0.045	0.088
	Red	-1.010	-0.989	-0.021	0.239
	Indian				
	Brown	-0.017	0.043	-0.060	0.091
	Green	0.297	0.289	0.009	0.549
100 kb windows					
	Chinese				
	Blue	-0.956	-0.907	-0.049	0.014*
	Purple	-0.784	-0.740	-0.045	0.015
	Red	-1.017	-0.993	-0.024	0.070
	Indian				
	Brown	-0.027	0.053	-0.080	0.015*
	Green	0.273	0.302	-0.029	0.276

Table S7. Upper F_{ST} outliers in each pairwise comparison in each of the four datasets.

M. mulatta from China	
Red : Blue	TARS2, TIMMDC1, ATP5O, ATP5J2, MRPS36, MRPL52, NDUFB8, MRPL51, ACP1, MRPL11, MRPL49, SURF1, MRPL10, MRPL57, MRPL54, PET100, MRPL4, NDUFA13, MRPS34, COX4I1
Red : Purple	MRPL24, ATP5O, MRPS36, CD14,NDUFA2, HARS2, TMEM70, TYMP, BCS1L, SURF1, MRPL10, POLRMT, MRPL54, SARS2,MRPS12, MRPL28, MRPS34, EARS2
Blue : Purple	DARS2, ATP5O, ATP5J2, NDUFB2, MRPS18A, ATP5I, LYRM7, CD14,NDUFA2 COX5A, MRPL52, TFAM, NDUFB8, MRPL43, TYMP, NDUFS1, ACP1, UQCC3, ATP5L, SURF1, MRPS7, UQCR, MRPL54, NDUFA7, MRPL4, MRPL28, MRPS34, COX4I1 , ATP5O, NDUFB2, MRPS18A, ATP5I, LYRM7, CD14, NDUFA2, NDUFAF1, COX5A, MRPL52, TFAM, NDUFB8, MRPL43, TYMP, ACP1, UQCC3, ATP5L, SURF1, MRPS7, UQCR, MRPL54, NDUFA7, MRPL4, MRPS34, COX4I1
M. mulatta from India	
Brown : Orange	MRPL3, UQCRQ, NDUFB8, MRPL44, MRPL30, MRPL49, FOXRED1, MRPL54,

	SARS2,MRPS12
Orange : Red	LARS2, ATP5MF, TMEM70, CYC1, NDUFB8, SARS2,MRPS12, MRPL28, MRPS34, COX4I1
Brown : Red	LARS2, ACAD9, ATP5MF, NDUFB8, SARS2,MRPS12, MRPL28, MRPS34, COX4I1
M. arctoides dataset	
M arctoides : M. assamensis	DARS2, NDUFAF3, ATP5J2, ATP5I, TMEM70, MRPS16, MRPL40, TYMP, ATP5B, NDUFB3, UQCC3, TTC19, POLRMT, NDUFA13, MRPL28
M. arctoides : M. fascicularis	TARS2, MRPL55, NDUFAF3, ATP5J, AARS2, RARS2, CD14, NDUFA2, HARS2, CYC1, MRPS16, TYMP, MRPL51, ATP5B, UQCC3, NDUFA13
M. arctoides : M. mulatta	MRPL55, ATP5J2, CD14, NDUFA2, HARS2, NDUFB8, MRPL43, MRPL51, ATP5B, UQCC3, TMEM126B, MRPL34
M. arctoides : M. thibetana	ATP5I, HARS2, MRPL51, UQCC3, MRPS7, NDUFA7, NDUFA13, MRPL28, MRPS34
M. assamensis : M. fascicularis	MRPL55, NDUFAF3, MRPL2, CD14, NDUFA2, HARS2, MRPS16, MRPL43, USMG5, ATP5B, UQCC3, MRPL10, MRPL57, POLRMT
M. assamensis : M. mulatta	ATP5F1, MRPL55, DARS2, NDUFAF3, MRPL2, MRPS36, UQCRQ, HARS2, NDUFB1, MRPS16, MRPL43, USMG5, ATP5B, UQCC3, TMEM126B, MRPL45
M. assamensis : M. thibetana	MRPL2, ATP5I, MRPS18C, NDUFAF2, HARS2, TMEM70, MRPS16, MRPL43, MRPL40, UQCC3, MRPS2, CCDC56, MRPS7, MRPL38, MRPL57, POLRMT, MRPL20, MRPS34
M. mulatta : M. fascicularis	MRPL37, NDUFAF3, ATP5J2, MRPL2, MRPL1, NDUFC1, MRPS36, LYRM7, CD14, NDUFA2, MRPL22, MRPS16, NDUFB8, MRPL43, USMG5, MRPL51, ATP5B, BCS1L, COX7C, TMEM126B, MRPL10, NDUFA13
M. thibetana : M. fascicularis	MRPL37, NDUFAF3, MRPL2, CD14, NDUFA2, HARS2, CYC1, MRPL43, USMG5, ATP5B, MRPL49, UQCC3, MRPL10, MRPS7, POLRMT, NDUFA13
M. thibetana : M. mulatta	ATP5F1, MRPL9, MRPL55, MRPL2, CD14, NDUFA2, HARS2, NDUFB8, MRPL43, NDUFV1, MRPL49, UQCC3, TMEM126B, MRPL34, NDUFA13
M. f. aurea dataset	
M. f. aureus : M. thibetana	MRPS21, NDUFAF3, ATP5MF, MRPL14, UQCRQ, MRPL52, BCS1L, UQCC3, MRPL10, CCDC56
M. f. aureus : M. fascicularis	MRPS21, HIGD1A, NDUFAF3, RARS2, UQCRQ, COX16, NDUFB8, MARS2, NDUFB3, BCS1L, MRPL53, MRPL23, NARS2, TMEM126B, TTC19, ATPAF2, MRPL10, CCDC56, TACO1
M. f. aureus : M. assamensis	MRPS21, NDUFAF3, ATP5MF, TFB1M, RARS2, MRPS18A, UQCRQ, BCS1L, UQCC3, TTC19, MRPL10, MRPL12, CARS2, NDUFA13
M. assamensis : M. thibetana	MRPL20, NDUFAF3, COX19, ATP5MF, RARS2, ATP5ME, MRPL52, CYC1, MRPL21, CARS2, UQCR, NDUFA7, NDUFA13, MRPL28, MRPS34
M. fascicularis : M. thibetana	NDUFAF3, MRPL2, UQCRQ, NDUFA2, CD14, HARS2, CYC1, BCS1L, COX7C, MRPL49, MRPL10, CCDC56, NDUFA13
M. fascicularis : M. assamensis	PDC, NDUFAF3, RARS2, MRPL2, UQCRQ, NDUFA2, CD14, HARS2, COX6C, CYC1, BCS1L, COX7C, MRPL49, UQCC3, NARS2, MRPL10, MRPL45, TACO1, NDUFA13

Table S8. Lower π outliers for each population or species in each of the four datasets.

M. mulatta from China	
Red	MRPL9, MRPL55, ATP5J2, UQCRQ, ATP5S, NDUFB8, MRPL43, MRPL53, MRPL49, TMEM126B, MRPL10, NDUFA7
Blue	MRPL55, ATP5J2, HARS2, NDUFB8, MRPL43, MRPL53, MRPL49, TMEM126B, NDUFA7
Purple	MRPL55, ATP5J2, UQCRQ, HARS2, NDUFB8, MRPL43, MRPL53, NDUFV1, MRPL49, TMEM126B, NDUFA7
M. mulatta from India	
Orange	MRPS21, MRPL18, MRPL2, UQCRQ, ATP5MPL, NDUFB8, MRPL43, MRPL10
Brown	MRPS21, MRPL18, MRPL2, UQCRQ, ATP5MPL, MRPL43, NDUFS1, UQCC3, MRPL10, MRPS7
Red	ATP5MF, MRPL2, UQCRQ, ATP5MPL, TMEM70, NDUFB8, LOC716161, SARS2, MRPS12, MRPL28, MRPS34, EARS2, COX4I1
M. arctoides dataset	
M. arctoides	MRPL55, ATP5I, TYMP, MRPL51, MRPS34
M. fascicularis	MRPL37, MRPL2, CD14, NDUFA2, HARS2, CYC1, MRPL43, USMG5, MRPL49, UQCC3, MRPL10, POLRMT, NDUFA13
M. mulatta	MRPL37, ATP5F1, MRPL9, MRPL55, ATP5J2, MRPL2, CD14, NDUFA2, HARS2, NDUFB1, NDUFB8, MRPL43, USMG5, NDUFV1, TMEM126B, MRPL50, MRPL34
M. thibetana	MRPL2, MRPL49, UQCC3, SURF1, CCDC56
M. assamensis	NDUFS5, MRPS21, MRPL55, DARS2, MRPL2, UQCRQ, MRPL15, TMEM70, MRPS16, MRPL43, MRPS26, NDUFB3, BCS1L, UQCC3, TTC19, POLRMT
M. f. aurea dataset	
M. f. aurea	MRPS21, NDUFAF3, ATP5MF, UQCRQ, MRPL43, MRPL51, BCS1L, UQCC3, MRPL10, CCDC56, CARS2
M. fascicularis	NDUFAF3, MRPL18, RARS2, MRPL2, NDUFAF2, MRPS36, MRPS27, NDUFA2, CD14, NDUFAF6, CYC1, BCS1L, MRPL53, COX7C, MRPL49, NARS2, TMEM126B, MRPL10, CCDC56, NDUFA13, NDUFB10
M. thibetana	MRPL18, MRPL2, NDUFA2, CD14, MRPL49, LOC722212, MRPL10, CCDC56, NDUFB10
M. assamensis	ATP5MF, MRPL36, NDUFS6, UQCC3, MRPL12, CARS2, POLRMT, NDUFA13, MRPS34, NDUFB10

Table S9. The rate ratio of nonsynonymous to synonymous substitutions per site was individually significantly higher in $N_{interact}$ than non-$N_{interact}$ in two of four focal taxa. The observed difference in the mean dN/dS ratio of $N_{interact}$ and non-$N_{interact}$ genes (Observed) is positive for all comparisons. P is the proportion of permutations that had a difference between randomized means that was greater than the observed, with asterisks indicating individual significance. n($N_{interact}$) and n(non-$N_{interact}$) are the numbers of genes that were included in the analysis after quality control.

Taxon	Observed	*P*	n($N_{interact}$)	n(non-$N_{interact}$)
M. mulatta from China	0.046	0.115	156	2722
M. mulatta from India	0.042	0.093	157	2651
M. arctoides dataset	0.226	0.001*	134	2566
M. f. aurea dataset	0.128	0.016*	162	2699

Table S10. $N_{interact}$ windows that were upper F_{ST} and lower π outliers for each dataset.

F_{ST} comparison	π population	# π outliers	# F_{ST} outliers	# shared outliers	π proportion shared	F_{ST} proportion shared	Gene acronyms of shared outliers
M. mulatta from China							
Red : Blue	Red	12	19	3	0.25	0.16	MRPL49; NDUFB8; MRPL10
Red : Blue	Blue	9	19	2	0.22	0.11	MRPL49; NDUFB8
Red : Purple	Red	12	19	1	0.08	0.05	MRPL10
Red : Purple	Purple	11	19	1	0.09	0.05	HARS2
Blue : Purple	Blue	9	26	3	0.33	0.12	MRPL43; NDUFA7; NDUFB8
Blue : Purple	Purple	11	26	3	0.27	0.12	MRPL43; NDUFA7; NDUFB8
M. mulatta from India							
Brown : Orange	Brown	10	11	1	0.10	0.09	UQCRQ
Brown : Orange	Orange	8	11	2	0.25	0.18	NDUFB8; UQCRQ
Brown : Red	Brown	10	10	0	0.00	0.00	
Brown : Red	Red	13	10	8	0.62	0.80	SARS2; ATP5MF; MRPS12; LOC716161; MRPS34; MRPL28; NDUFB8; COX4I1
Orange : Red	Orange	8	11	1	0.13	0.09	NDUFB8
Orange : Red	Red	13	11	8	0.62	0.73	TMEM70; SARS2; ATP5MF; MRPS12; MRPS34; MRPL28; NDUFB8; COX4I1
M. arctoides dataset							
M arctoides : M. assamensis	*M. arctoides*	5	15	2	0.40	0.13	TYMP; ATP5I
M. arctoides : M. assamensis	*M. assamensis*	16	15	7	0.44	0.47	TTC19; TMEM70; POLRMT; NDUFB3; UQCC3; MRPS16; DARS2
M. arctoides : M. fascicularis	*M. arctoides*	5	16	3	0.60	0.19	MRPL51; TYMP; MRPL55

M. arctoides : M. fascicularis	*M. fascicularis*	13	16	6	0.46	0.38	NDUFA13; CYC1; UQCC3; CD14; HARS2; NDUFA2
M. arctoides : M. mulatta	*M. arctoides*	5	12	2	0.40	0.17	MRPL51; MRPL55
M. arctoides : M. mulatta	*M. mulatta*	17	12	9	0.53	0.75	MRPL34; ATP5J2; NDUFB8; MRPL55; CD14; MRPL43; TMEM126B; HARS2; NDUFA2
M. arctoides : M. thibetana	*M. arctoides*	5	9	3	0.60	0.33	MRPL51; MRPS34; ATP5I
M. arctoides : M. thibetana	*M. thibetana*	5	9	1	0.20	0.11	UQCC3
M. assamensis : M. fascicularis	*M. assamensis*	16	14	6	0.38	0.43	POLRMT; MRPL2; UQCC3; MRPL55; MRPS16; MRPL43
M. assamensis : M. fascicularis	*M. fascicularis*	13	14	9	0.69	0.64	MRPL10; POLRMT; USMG5; MRPL2; UQCC3; CD14; MRPL43; HARS2; NDUFA2
M. assamensis : M. mulatta	*M. assamensis*	16	16	7	0.44	0.44	UQCRQ; MRPL2; UQCC3; MRPL55; MRPS16; MRPL43; DARS2
M. assamensis : M. mulatta	*M. mulatta*	17	16	8	0.47	0.50	ATP5F1; USMG5; MRPL2; MRPL55; NDUFB1; MRPL43; TMEM126B; HARS2
M. assamensis : M. thibetana	*M. assamensis*	16	18	6	0.38	0.33	TMEM70; POLRMT; MRPL2; UQCC3; MRPS16; MRPL43
M. assamensis : M. thibetana	*M. thibetana*	5	18	3	0.60	0.17	MRPL2; CCDC56; UQCC3
M. mulatta : M. fascicularis	*M. mulatta*	17	22	9	0.53	0.41	MRPL37; ATP5J2; USMG5; MRPL2; NDUFB8; CD14; MRPL43; TMEM126B; NDUFA2
M. mulatta : M. fascicularis	*M. fascicularis*	13	22	8	0.62	0.36	MRPL10; MRPL37; NDUFA13; USMG5; MRPL2; CD14; MRPL43; NDUFA2
M. thibetana : M. fascicularis	*M. thibetana*	5	16	3	0.60	0.19	MRPL49; MRPL2; UQCC3

	M. thibetana : M. fascicularis	*M. fascicularis*	13	16	13	1.00	0.81	MRPL49; MRPL10; POLRMT; MRPL37; NDUFA13; USMG5; MRPL2; CYC1; UQCC3; CD14; MRPL43; HARS2; NDUFA2
	M. thibetana : M. mulatta	*M. thibetana*	5	15	3	0.60	0.20	MRPL49; MRPL2; UQCC3
	M. thibetana : M. mulatta	*M. mulatta*	17	15	12	0.71	0.80	ATP5F1; MRPL34; NDUFV1; MRPL2; MRPL9; NDUFB8; MRPL55; CD14; MRPL43; TMEM126B; HARS2; NDUFA2
M. f. aurea dataset								
	M. f. aureus : M. thibetana	*M. f. aureus*	11	10	8	0.73	0.80	MRPL10; MRPS21; ATP5MF; CCDC56; NDUFAF3; UQCRQ; UQCC3; BCS1L
	M. f. aureus : M. thibetana	*M. thibetana*	9	10	2	0.22	0.20	CCDC56; MRPL10
	M. f. aureus : M. fascicularis	*M. f. aureus*	11	19	6	0.55	0.32	MRPL10; MRPS21; CCDC56; NDUFAF3; UQCRQ; BCS1L
	M. f. aureus : M. fascicularis	*M. fascicularis*	21	19	8	0.38	0.42	MRPL10; CCDC56; RARS2; NDUFAF3; NARS2; TMEM126B; BCS1L; MRPL53
	M. f. aureus : M. assamensis	*M. f. aureus*	11	14	8	0.73	0.57	CARS2; MRPL10; MRPS21; ATP5MF; NDUFAF3; UQCRQ; UQCC3; BCS1L
	M. f. aureus : M. assamensis	*M. assamensis*	10	14	5	0.50	0.36	CARS2; ATP5MF; NDUFA13; UQCC3; MRPL12
	M. assamensis : M. thibetana	*M. assamensis*	10	15	4	0.40	0.27	CARS2; NDUFA13; MRPS34; ATP5MF
	M. assamensis : M. thibetana	*M. thibetana*	9	15	0	0.00	0.00	
	M. fascicularis : M. thibetana	*M. fascicularis*	21	13	11	0.52	0.85	MRPL49; MRPL10; COX7C; NDUFA13; CCDC56; NDUFAF3; MRPL2; CYC1; CD14; BCS1L; NDUFA2
	M. fascicularis : M. thibetana	*M. thibetana*	9	13	6	0.67	0.46	MRPL49; MRPL10; CCDC56; MRPL2; CD14; NDUFA2

M. fascicularis : M. assamensis	*M. fascicularis*	21	18	12	0.57	0.67	MRPL49; MRPL10; COX7C; RARS2; NDUFA13; NDUFAF3; MRPL2; CYC1; CD14; NARS2; BCS1L; NDUFA2
M. fascicularis : M. assamensis	*M. assamensis*	10	18	2	0.20	0.11	NDUFA13; UQCC3

Table S11. Results of analyses of introgression using Patterson's D statistic. Analyses were performed for the *M. arctoides* and the *M. f. aurea* datasets and using 30 or 100kb genomic windows. Values of Patterson's D are presented for the entire genome (Genome-wide), for $N_{interact}$ and non- $N_{interact}$ windows, and for non-genic windows based on configurations of taxa (P1:P2:P3:O) with species abbreviated using the first three letters of the species name. The test statistic for permutation tests is the difference between the mean Patterson's D statistic in $N_{interact}$ and non- $N_{interact}$ windows (Difference) and an asterisk indicates an individually significant P values (P) based on a permutation test.

M. arctoides dataset							
Window size	P1: P2: P3: O	Genome-wide	$N_{interact}$ windows	non-$N_{interact}$ windows	Non-genic windows	Difference	P
30k	arc:ass:mul:nem	-0.104	-0.038	-0.100	-0.105	0.062	0.907
	arc:thi:mul:nem	-0.115	0.017	-0.021	-0.115	0.133	0.989
100k	arc:ass:mul:nem	-0.124	-0.122	-0.119	-0.128	-0.003	0.428
	arc:thi:mul:nem	-0.114	-0.074	-0.112	-0.115	0.038	0.857
M. f. aureus dataset							
30k	fas:aur:ass:nem	0.084	0.057	0.079	0.086	-0.021	0.758
	fas:aur:thi:nem	0.083	0.071	0.078	0.084	-0.007	0.599
100k	fas:aur:ass:nem	0.103	0.079	0.096	0.107	-0.017	0.692
	fas:aur:thi:nem	0.102	0.074	0.096	0.106	-0.023	0.758
